# *Enterococcus* spp. as a Producer and Target of Bacteriocins: A Double-Edged Sword in the Antimicrobial Resistance Crisis Context

**DOI:** 10.3390/antibiotics10101215

**Published:** 2021-10-07

**Authors:** Ana C. Almeida-Santos, Carla Novais, Luísa Peixe, Ana R. Freitas

**Affiliations:** 1 UCIBIO–Applied Molecular Biosciences Unit, REQUIMTE, Laboratory of Microbiology, Department of Biological Sciences, Faculty of Pharmacy, University of Porto, 4050-313 Porto, Portugal or up201404762@up.pt (A.C.A.-S.); casilva@ff.up.pt (C.N.); 2 Associate Laboratory i4HB, Institute for Health and Bioeconomy, Faculty of Pharmacy, University of Porto, 4050-313 Porto, Portugal; 3 TOXRUN–Toxicology Research Unit, Department of Sciences, University Institute of Health Sciences, CESPU, CRL, 4585-116 Gandra, Portugal

**Keywords:** antimicrobial resistance, alternatives to antibiotics, bacteriocins, multidrug-resistant infections, enterococci, enterocins, VRE, gut microbiota

## Abstract

*Enterococcus* spp. are one of the most frequent producers of bacteriocins (enterocins), which provides them with an advantage to compete in their natural environment, which is the gut of humans and many animals. The enterocins’ activity against microorganisms from different phylogenetic groups has raised interest in *Enterococcus* spp. in different contexts throughout the last decades, especially in the food industry. Nevertheless, some species can also cause opportunistic life-threatening infections and are frequently multidrug-resistant (MDR). Vancomycin-resistant *Enterococcus* (VRE), in particular, are an ongoing global challenge given the lack of therapeutic options. In this scenario, bacteriocins can offer a potential solution to this persistent threat, either alone or in combination with other antimicrobials. There are a handful of studies that demonstrate the advantages and applications of bacteriocins, especially against VRE. The purpose of this review is to present a current standpoint about the dual role of *Enterococcus* spp., from important producers to targets needed to be controlled, and the crucial role that enterocins may have in the expansion of enterococcal populations. Classification and distribution of enterocins, the current knowledge about the bacteriocinome of clinical enterococci, and the challenges of bacteriocin use in the fight against VRE infections are particularly detailed.

## 1. Introduction

The number of bacterial strains resistant to antibiotics has increased radically, making antimicrobial resistance (AMR) a major global health emergency of the 21st century [1,2]. Currently, AMR infections are responsible for at least 700,000 deaths each year worldwide (https://www.who.int/news/item/29-04-2019-new-report-calls-for-urgent-action-to-avert-antimicrobial-resistance-crisis, accessed on 25 May 2021). If no action is taken, this figure could increase up to 10 million by the year 2050, with a global financial burden of USD 100 trillion [2]. AMR is so alarming that the World Health Organization (WHO) just stated that combating AMR is one of the top 10 global health issues to track in 2021 by identifying its causes, routes of transmission, and encouraging the development of new antimicrobials (https://www.who.int/news-room/spotlight/10-global-health-issues-to-track-in-2021, accessed on 25 May 2021). Several factors contribute to the AMR crisis. The main drivers include antibiotics misuse and overuse by the general population and in food-producing animals, as well as their incorrect and indiscriminate administration by healthcare professionals [3,4]. As a consequence of these malpractices, resistance to the so-called “last-resort” antibiotics is not an emerging problem anymore, but a reality [5,6]. Hence, there is a crucial need for the development of new antimicrobials, which, if not pursued, in the worst-case scenario, could result in even common procedures, such as dental treatments, becoming life threatening. For these reasons, a number of international health agencies (e.g., European Medicines Agency, Food and Drug Administration, World Health Organization, European Center for Disease prevention and Control) support the development of new antimicrobials, treatments, and preventive approaches [7].

Alternatives, such as bacteriophages, probiotics, lysins, antimicrobial peptides, antibodies, and vaccines, have been investigated to reduce the use of antibiotics [8]. An emerging area of interest is the development of very-narrow-spectrum or species-specific antimicrobials, where the goal is to target only the microbe(s) that are actually causing infection, also limiting the selection for resistance and dysbiosis commonly induced in the coexisting beneficial microbiota by broad-spectrum antimicrobials [9]. Such an approach can include the use of bacteriocins, which are a subset of the antimicrobial peptides (AMPs) that are produced by bacteria [10]. They are proteins or peptides, usually ribosomally synthesized, displaying antimicrobial activity towards strains of the same or other bacterial species, with an activity that is influenced by different environmental factors, such as temperature and pH, among others [11,12]. While some bacteriocins only exhibit antimicrobial activity against bacteria of the same species or against close phylogenetic groups, designated as narrow-spectrum bacteriocins, others are active against a variety of genera and, thus, display a broad antimicrobial activity. In this manner, bacteriocins are extremely diverse in structure, being encoded by complex and variable gene clusters, which go through rapid evolution and recurrent horizontal transfer, namely by plasmids [13]. These bacteriocin gene clusters usually encode the bacteriocin itself, but also biosynthetic enzymes, export and immunity mechanisms, and even quorum sensing bacteriocin production regulators. Bacteriocin producers usually protect themselves through self-immunity proteins and/or by efflux transporters [13]. This protection is generally restricted to the bacteriocin itself or very closely related ones.

Even though bacteriocins are being explored for diverse medical approaches (in veterinary medical products; prevention of biofilm formation in medical device surfaces, such as urinary catheters; anti-inflammatory or anticancer substances) [14,15,16], their application is mostly associated with their antimicrobial activity [10]. The mechanisms behind their activity, which are not fully understood, are diverse and include the disruption of the cell membranes by pore formation (e.g., enterocin AS-48), and the impairment of cell wall synthesis (e.g., nisin) or of nucleic acid replication and translation (e.g., microcin B17) [13]. In contrast with antibiotics, which, to different extents, decrease bacterial diversity, generating opportunities for the overgrowth of pathogenic bacteria, and can create conditions for the development of antibiotic resistance, the action of bacteriocins can lead to different outcomes by promoting or preventing invasion by a new bacterial strain: (a) they may be an advantage to an invasive bacterial strain, allowing it to clear the resident community of bacteriocin-sensitive bacteria; (b) if produced by the resident community, they can act as a shield, preventing the colonization by a bacteriocin-susceptible invader (colonization resistance), although susceptible bacteria are not necessarily eradicated from a specific niche if they are spatially segregated from bacteriocin producers; and (c) bacteriocins can act as signaling peptides/quorum-sensing molecules in the complex gut network [13,17]. Clearly, bacteriocin production is a means to outcompete with other bacteria in mixed communities sharing limited nutrients, space, or adhesion sites to avoid clearance [13]. Moreover, when a bacteriocin is able to kill the competitive bacteria by lysis, it promotes the release of intracellular components, used as essential nutrients, as well as DNA that can be acquired by transformation events contributing to genomic diversity [13]. The fitness of a certain bacterial strain that is competing with bacteriocin-susceptible bacteria can be shaped by gain, loss, or diversification of bacteriocin genes. Nevertheless, a bacteriocin only generates a considerable fitness benefit while inhibiting relevant competitors but avoiding damage to dependent mutualists (for example, providing common nutrients) if the greater competitors are not able to develop resistance and if the fitness benefit is higher than the production metabolic cost [13]. This is a delicate balance, probably affected by yet unknown factors, between antagonistic and mutualistic strains shaping the human microbiome. The knowledge about the role of bacteriocins in this complex network is still in its infancy, even with current metagenomic approaches, as these may miss spatial distribution of strains within a community [13].

Bacteriocins are predominantly produced by bacteria from the *Firmicutes* phylum, in particular, the lactic acid bacteria (LAB) group of bacteria. Among this group, *Enterococcus* spp. are well known as one of the greatest bacteriocin producers, both in number of isolates and diversity of enterocins. They are part of our intestinal microbiota and often accumulate multiple adaptive features to diverse environmental conditions and hosts [18]. It was suggested that bacteriocin production ensures niche competition of enterococci within the gut complex microbial network, namely against particular pathogens [18]. However, enterococci, mainly the clinically relevant multidrug-resistant *Enterococcus faecium* and *Enterococcus faecalis*, can also be associated with life-threatening infections [19]. Indeed, vancomycin-resistant *E. faecium* (VREfm) stands as one of the most common causes of hard-to-treat hospital infections and, due to lack of therapeutic options, is recognized by WHO as a priority pathogen urgently requiring new antimicrobials [20], with the use of next-generation probiotics derived from the gut microbiota and bacteriocins as alternatives proposed [21]. Within this context, enterococci can themselves be included in the list of probiotics with the selectivity of their enterocins being a topic of interest to be better addressed. The bacteriocin sensitivity of a given strain can depend on the presence of immunity genes related to its own bacteriocin production (same operon), the presence of extra immunity genes, and other general strain features, such as the membrane composition or the physicochemical properties of the peptide [22].

This review aims to document the current standpoint about the dual role of *Enterococcus* spp. within the bacteriocin complex context, from important producers to targets that need to be controlled, during infection and colonization scenarios. Classification of enterocins and their spectrum activity, the challenges of using bacteriocins in the fight against VRE and MDR infections in general, alongside the crucial role that enterocins may have in the expansion of enterococcal populations and their mobile genetic elements are here discussed.

## 2. Diversity of Bacteriocins Produced by Enterococci

**Figure 1:**
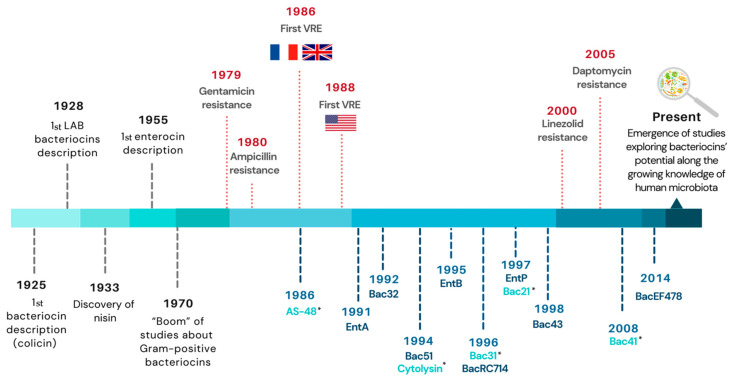
Timeline of important events in the history of bacteriocins. Enterocins specifically described in clinical enterococci isolates so far are included as blue lines: dark blue indicates enterocins produced by *E. faecium* and light blue by *E. faecalis*; the years indicated correspond to the first mention of a given enterocin, either the year of isolation or, whenever unknown, the year of bacteriocin description/publication (marked with * in the latter case). The year corresponding to the appearance of the most clinically relevant antibiotic resistance in enterococci appear as red lines. Abbreviations: LAB, lactic acid bacteria; VRE, vancomycin-resistant enterococci; Bac, bacteriocin; Ent, enterocin.

It was not until 1952 that the term “bacteriocine” emerged, and it is still used nowadays (without the final “e”) [23]. Until the 1970s, most studies were focused on Gram-negative bacteria [24], but, since then, numerous reports on Gram-positive bacteria have emerged given their special bacteriocin production ability and the increasing understanding of bacteriocin’s applications, either in food preservation or in the treatment of bacterial infections [25,26] (Figure 1). In enterococci, a bacteriocin-like activity was first described in 1955, in both *E. faecalis* and *E. faecium* [27], and, in 1963, the term “enterocin” was first used in a publication [28] to designate a type of bacteriocin that is synthesized by *Enterococcus* (at that time *Streptococcus*).

Descriptions of enterococci strains as producers of new enterocins exhibiting activity against different bacterial species are frequent, with multiple examples being available in the literature [18]. Genes encoding enterocins have been identified in enterococci strains from a variety of sources and hosts, which suggests a role of enterocins in their adaptation to environments suffering variable challenges [18,29]. Moreover, their frequent location on conjugative or mobilizable plasmids, along with genes encoding other adaptive features (e.g., antibiotic resistance, metal tolerance, virulence factors) and toxin/anti-toxin systems, reinforces the importance of these multiple-featured plasmids for enterococci competition and survival [18,30,31]. On another hand, bacteriocin production can correspond to a transient trait after acquisition by a bacterial strain through horizontal gene transfer in response to a need within a certain microbial community and niche [13].

Enterocins have been particularly explored against foodborne pathogens or food deterioration species, with most studies about this topic focusing their interest on food biopreservation, increase in food shelf-life extension, or improvement of animals’ health [32,33]. However, in the last years, the interest in enterocins has also been focused on decolonizing agents of antibiotic-resistant enterococci from patients’ gut to prevent the emergence of resistant enterococcal infections that are otherwise difficult, or impossible, to treat [26,34]. Recent in vivo studies showed that enterocins producing enterococci could outcompete with other enterococci, namely VRE, without a significant disruption of the general microbiota, as well as be involved in the active conjugation of plasmids carrying bacteriocin-encoding genes, enhancing the number of bacteriocin producers and eliminating bacteriocin susceptible populations [13,35]. However, for most enterocins, studies have mainly been using in vitro models and included a few enterocin target bacteria [36,37], with the real impact of their use in the modulation of the complex microbiota network of humans and animals remaining underexplored.

Bacteriocins have been associated with the concepts: *probiotics* and *postbiotics*. The first refers to “live microorganisms that, when administered in adequate amounts, confer a health benefit on the host”, whereas a postbiotic is a “preparation of inanimate microorganisms and/or their components that confers a health benefit on the host” [38]. Probiotics, including enterococci-containing ones, have been associated with intestinal health improvement, strengthening of the immune response, and infection prevention. Numerous probiotic formulations contain bacteriocin-producing strains, which seems to be an important criterion upon the selection of a probiotic strain [17]. It has been hypothesized that the benefits of probiotics, namely to enhance the beneficial bacteria in the human gut and to fight pathogens, are due to the production of bacteriocins, but such dynamics are still not completely understood and bacteriocins are often not disclosed within probiotic cocktails. The impact of enterocins in food safety and preservation, as well as against antibiotic-resistant bacteria, along with the concomitant tolerance of enterococci to pH, bile salts, and other physicochemical factors, position enterocin-producing strains as tempting candidates to be used as food additives and probiotics [39]. Enterococci have been used safely as probiotics in humans or as food additives in livestock for decades, as long as there is a clear distinction between pathogenic and nonpathogenic strains [38,39]. Enterocins could also be considered for use as postbiotics products, despite the need for regulatory processes, as new drugs undergo if they are intended for therapeutic use or for addition to food products [38]. In this section, we summarize the main known classes of bacteriocins produced by enterococci.

### 2.1. Classification and Origin of Known Enterocins

Due to its diversity and complexity, there have been different classification systems for bacteriocins throughout the years, mainly for LAB-produced ones. Klaenhammer, in 1993, was the first to propose a classification scheme for LAB bacteriocins based in four classes [40], whereas others opted for only two classes [41]. Although bacteriocin classification is still a controversial matter, there are two well-defined major classes for enterocins. Class I includes the lantibiotics and Class II comprises the unmodified non-lantibiotics [18,25]. Each class is categorized into various subclasses, but inconsistency between classifications is especially noticed for those. Class III either includes bacteriolysins [10,25] or cyclic peptides [32] according to different studies. In those only considering two classes, the bacteriolysins are simply a separated category [41]. Enterocins from Class I and II are able to cause cell death mainly by pore formation in the cell membrane of the target bacteria after binding to specific receptors (e.g., lipid II in the case of lantibiotics, mannose phosphotransferase system (Man-PTS) for non-lantibiotics, among others), whereas class III bacteriolysins cause cell death by cleaving the peptidoglycan cross-links of the target cell wall [18,42].

The detailed analysis we performed here about the main known bacteriocins produced by enterococci (Table S1) allowed us to observe: (i) an equilibrated distribution of bacteriocins between *E. faecium* (*n* = 19 bacteriocins) and *E. faecalis* (*n* = 24 bacteriocins) species; (ii) *E. faecium*/*E. faecalis* as main producers of different bacteriocins in comparison to other enterococcal species (*n* = 8); and (iii) a species-specificity of bacteriocin-encoding genes, since highly homolog bacteriocins were limited to a single enterococcal species. We speculate that such bacteriocin species specificity is probably associated with the plasmid pool usually observed within each species that is also species-specific (e.g., pheromone-responsive plasmids in *E. faecalis*) or with specific genomic events allowing the establishment of bacteriocins on the core genome (e.g., EntA in *E. faecium*, see below). In addition, some enterococci strains harbor simultaneously more than one bacteriocin-related gene (e.g., *E. faecium* L50 produces L50A/B, EntP, and EntQ, see below), which can provide them with a competitive advantage against other strains of the same or different species. In fact, it is currently known, mainly through genomic data, that a single enterococci strain can carry several different bacteriocin-encoding genes, although the bacteriocinome of specific enterococcal populations is still highly underexplored (discussed below). The term “bacteriocinome” is employed here to designate the genomic content of a given strain in bacteriocin-encoding genes.

#### 2.1.1. Class I—Lantibiotics

Lantibiotics are small peptides (<5 kDa) that are post-translationally modified to their biologically active forms, resulting in unusual amino acids, such as lanthionine residues [25,32]. These peptide modifications are advantageous by making bacteriocins more stable to the activity of proteases or to high temperatures and pH [10]. Furthermore, bacteriocins from this class are commonly highly effective against Gram-positive pathogens [43].

Cytolysin and enterocin W, both produced by *E. faecalis* strains, are the only two lantibiotic-type enterocins currently known [18]. Cytolysin can be encoded by genes located on the chromosome or in a pheromone-responsive plasmid (pAD1) and entails two peptides (CylL and CylS), both needed for antimicrobial activity [44,45]. Cytolysin is often referred to as hemolysin, since it is active against eukaryotic cells (e.g., erythrocytes and human intestinal epithelial cells), besides being active against a wide variety of Gram-positive bacteria [18,32] (Table S1). This hemolytic phenotype is especially found among clinical isolates and may contribute to the severity of numerous diseases [46,47,48]. Due to its virulence-associated feature, cytolysin would not be safe or recommended to be used as an antimicrobial agent.

Few data are available concerning enterocin W. The producing strain was isolated from a traditional Thai fermented fish and is also a two-peptide lantibiotic. Enterocin W is active against different Gram-positive species, such as *Bacillus coagulans*, *B. circulans*, *Listeria innocua*, and *E. faecalis*, among others (Table S1), and its antimicrobial activity is greater with the combination of the two peptides (Wα and Wβ) [49].

#### 2.1.2. Class II—Non-Lantibiotics

Class II bacteriocins include the majority of described enterocins [18]. They are small (<10 kDa), heat-resistant, and do not undergo extensive post-translational modification as lantibiotics do. Although, there are some exceptions, such as the presence of disulfide bridges in some molecules (essential for antimicrobial activity) and when the leader peptide undergoes cleavage during the transport out of the cell [18,32,50]. Although their classification remains debatable, they are divided into several subclasses: class IIa (the pediocin-like), class IIb (two-peptides bacteriocins), class IIc (circular bacteriocins), and class IId (unmodified, linear, non-pediocin-like bacteriocins). The latter group also includes leaderless bacteriocins, which are oddly different from other ones of class II [18]. The biosynthesis of some bacteriocins from class II can be regulated by a quorum-sensing mechanism through a three-component regulatory system. Such a system consists of an inducing peptide, a histidine kinase sensor protein, and a response regulator (DNA-binding effector protein) [51].

##### Class IIa—The Pediocin-Like Bacteriocins

The antimicrobial activity of pediocin-like bacteriocins range from a narrow to a broad spectrum. They are especially potent against *Listeria* species, and particularly against the foodborne pathogen *Listeria monocytogenes* [50]. This fact is due to the presence of a conserved sequence (YGNGVXC) in the N-terminal region, allowing these bacteriocins to act at low nanomolar concentrations against *Listeria* species [18,52].

Enterocins belonging to this class have been identified in six enterococcal species: *E. faecium, E. faecalis, Enterococcus mundtii, Enterococcus avium, Enterococcus durans*, and *Enterococcus hirae* (Table S1). *E. faecium* is the most recurrent species found among Class IIa enterocin producers. Numerous examples of bacteriocins fall within this group, with most being identified in isolates from foodstuffs and dairy products, followed by animals and healthy or clinical human samples (Table S1). Particularly, enterocins A (EntA) and P (EntP) and bacteriocins 43, 31, and RC714 are among the best documented enterocins produced by clinical isolates (Table S1).

One of the commonest enterocins from the pediocin-like group is EntA. It has antimicrobial activity, mainly directed against *L. monocytogenes*, and is one of the most potent enterocins in this subgroup, possibly due to the presence of two disulfide bridges, one located in the N-terminal and the other in the C-terminal part [53,54]. EntA is produced by a variety of *E. faecium* strains from different sources (e.g., dry sausages, black olives, dairy sources, and Japanese rice-bran), being especially well documented in isolates recovered in different types of food [55] (Table S1). Although there is a clear lack of surveillance studies analyzing the distribution of this and other enterocins among enterococci, especially from clinical samples, Freitas et al. (2016) reported a high incidence of EntA in outbreak VREfm and vancomycin-susceptible *E. faecium* (VSEfm) obtained from hospitalized patients in different countries over the last decades [56]. Likewise, Strateva et al. (2015) reported a high incidence of EntA in clinical *E. faecium* isolates from various Bulgarian hospitals [57]. Indeed, EntA has been suggested to be part of the core genome of *E. faecium* [58], being chromosomally located, suggesting it may provide a competitive niche advantage to this species. *E. faecium* producing EntA often carry genes coding for other bacteriocins, such as enterocins B, P, L50, and Q [29,59,60].

EntP has been identified either on the chromosome or plasmids of different *E. faecium* strains isolated from foodstuffs or clinical isolates (Table S1) [61,62,63]. It has a wide antimicrobial spectrum that includes foodborne pathogens, such as *L. monocytogenes, Clostridium botulinum, Clostridium perfringens*, and *Staphylococcus aureus*, along with spoilage bacteria *Staphylococcus carnosus*, *Clostridium sporogenes*, *E. faecalis*, and *Propionibacterium* species, and even VREfm strains (Table S1) [61,63]. A broad antimicrobial spectrum alongside other characteristics, such as thermal resistance, activity in a wide range of pH values, and sensitivity to proteolytic enzymes [61], make this enterocin a potential food preservative. Yet, a few studies also reported the presence of EntP in clinical isolates, although at low rates. Freitas et al. (2016) detected the presence of EntP in VREfm and VSEfm isolates obtained from hospitalized patients in different countries, whereas Strateva et al. (2015) identified EntP production in isolates from inpatients and outpatients from Bulgarian hospitals, suggesting it could contribute to enterococci virulence or colonization ability of the human host [56,57].

Bacteriocin 43 (Bac43) was firstly described in VREfm strains recovered from hospitalized patients in the USA during the 1990s. Bac43 was also identified in one fecal sample from a healthy Japanese medical student [64]. It has antimicrobial activity against *L. monocytogenes, E. hirae, E. durans, E. faecalis*, and *E. faecium* strains and has been described on small mobilizable plasmids [56,64]. Data from Todokoro, et al. (2006) study suggested that pDT1-like plasmids have spread among different clinical VREfm strains according to their PFGE patterns [64]. Freitas et al. (2016) also detected Bac43, after 1998, on small theta-replicating plasmids (around 7 kb) of different VREfm strains from hospitalized patients in Germany and Canada, even though it was very rare [56]. This observation suggests that Bac43 occurrence may be influenced by clonal expansion and/or correspond to a more recent acquisition.

Bacteriocin RC714, isolated in a clinical VanA *E. faecium* RC714 strain in 1996, shares 98% identity with Bac43 [64]. According to available studies, this bacteriocin shows antimicrobial activity against vancomycin-susceptible, as well as VanA, *E. faecalis*, *E. faecium*, and *E. hirae* from human clinical/fecal and sewage samples. Moreover, it has inhibitory activity against *Lactobacillus plantarum, Lactobacillus paracasei, Pediococcus pentosaceus, Leuconostoc* spp., and several species of *Listeria* [65].

Bacteriocin 31 (Bac31) was described in a clinical *E. faecalis* YI717. Subsequent screening studies identified this bacteriocin in dairy *E. durans* isolates [66]. It is active against *L. monocytogenes*, *E. hirae* 9790, and clinical VREfm. The coding gene is located on a 57.5-kb pheromone-responsive conjugative plasmid pYI17 [67]. It differs from the other bacteriocins from class IIa in the N-terminal sequence (YGNGLXaaC), which raises the question if Bac31 should be considered a member of class IIa [68].

The remaining bacteriocins included in Table S1 have been less frequently detected, which can be due to the few studies assessing their distribution. They were described among samples obtained from humans, animals, several types of food, grass, or sewage (Table S1). Bacteriocins GM-1, avicin A, and T8 were identified in *E. faecium* or *E. avium* strains obtained from human samples. The three bacteriocins show antimicrobial activity against species of *Enterococcus* and *Listeria*, among others (Table S1). Different bacteriocins (MC4-1, hiracin JM79, S37, and E50-52) have been isolated in *E. faecalis*, *E. faecium*, or *E. hirae* from animal samples, with all displaying activity against *L. monocytogenes*, while activity against other bacterial species was variable. Enterocins CRL35, mundticin QU 2, mundticin ATO6, identified in *E. mundtii*, and durancin GL, from *E. durans*, were described in different food samples and presented activity at least against *Listeria* spp. (Table S1). Finally, mundticin KS and enterocin SE-k4, produced by *E. mundtii* and *E. faecalis*, respectively, were both sampled from grass silage in Thailand whereas enterocin M was found to be produced by an *E. faecium* strain isolated from sewage sludge, and exhibited activity against a different bacterial genus (Table S1).

##### Class IIb—Two-Peptide Bacteriocins

The two-peptide class IIb bacteriocins are composed of two different peptides, which are both needed in equal amounts in order to exert maximum antimicrobial activity. Although individual peptides can also confer antimicrobial activity, it is always slighter when compared to the combined peptides [18]. The enterocins included in the two-peptide group are enterocin C (EntC), enterocin 1071, and enterocin X from both *E. faecalis* and *E. faecium* species (Table S1).

EntC was identified in *E. faecalis* C901 strain obtained from human colostrum. It consists of two peptides, EntC1 and EntC2, and, as expected, both are needed for optimal antimicrobial activity. The bacteriocin genes are carried on a 9-kb-size plasmid, pEntC1, and have a broad spectrum of antibacterial activity against several Gram-positive bacteria, including *E. faecalis, E. faecium, Lactococcus lactis, Lactobacillus paracasei, Streptococcus anginosus*, among others [69].

Enterocin 1071 was described in *E. faecalis* strains isolated from minipig fecal and dairy samples [70,71]. Enterocin 1071 is composed of 1071A and 1071B peptides and its genes are encoded on a 50-kb conjugative plasmid. Its inhibitory spectrum includes various Gram-positive bacteria, such as *E. faecium* RC3, several *E. faecalis* strains, and *L. innocua* [70].

Enterocin X was first characterized in *E. faecium* KU-B5, a strain isolated from Thailand sugar apples. Interestingly, the antibacterial activity of both peptides, Xα and Xβ, was not always enhanced when the peptides were combined, varying according to the indicator strain. In fact, the activity of both peptides combined was only enhanced against strains of *E. faecium* and against different species of *Bacillus*, when compared with both peptides separately [72].

##### Class IIc—Circular Bacteriocins

Circular bacteriocins do not have free ends because the resulting N-terminal residue is covalently linked to the C-terminal residue, forming a circularized form [18]. Known circular enterocins, which are not commonly found, were identified in *E. faecalis* species, and only two of the three described to date have been found in clinical isolates (BacAS-48 and Bac21) (Table S1).

Bacteriocin AS-48 (BacAS-48) is one of the most studied enterocins. It was isolated from clinical *E. faecalis* S-48 of a human wound exudate, being encoded on a 56-kb pMB2-type conjugative pheromone-responsive plasmid. AS-48 is a broad-spectrum bacteriocin, being able to inhibit both Gram-negative (e.g., *Pseudomonas* spp.) and Gram-positive bacteria (e.g., *Bacillus* and *Enterococcus* species) [73,74].

Bacteriocin 21 (Bac21) was identified in an *E. faecalis* clinical isolate and encoded on a 59-kb pheromone-responsive conjugative plasmid, pPD1. Bac21 is at least active against a few species of *Streptococcus* (e.g., *S. agalactiae*, *S. sanguis*, and *S. aureus*), and *Enterococcus* (e.g., *E. faecalis*, *E. faecium*, and *E. hirae*) [75]. Interestingly, the highly transferable pheromone-like plasmids in which BacAS-48 and Bac21 are located, pMB2 and pPD1, respectively, respond to identical pheromones (cPD1-like), meaning that these bacteriocins can spread amongst different plasmid-free strains presenting the same pheromones [76]. It is also not surprising that these bacteriocins occur only among *E. faecalis*, given the narrow host spectrum of pheromone-responsive plasmids that are mostly restricted to this species [77].

Data about enterocin 4 are rather scarce. It is produced by *E. faecalis* INIA 4, a strain isolated from raw ewe’s milk, and its spectrum of antimicrobial activity includes various Gram-positive bacteria, such as *Clostridium tyrobutyricum, Lactobacillus buchneri, Lactobacillus brevis, L. monocytogenes, L. innocua, E. faecalis*, and *E. faecium* (Table S1) [78].

##### Class IId—Leaderless Bacteriocins

Leaderless bacteriocins are synthesized without a leader peptide, hence the designation, and can be composed by one (e.g., enterocins Q, EJ97, RJ-11, and DD14) or two peptides (e.g., enterocins 62-6, 7, MR10A/B, 7A/B, and L50) [18]. The vast majority of the bacteriocins from this group are isolated from enterococci of foodstuffs, but can also be found in isolates from healthy humans, animals, and wastewaters (Table S1). As far as we know, there has not been any description of leaderless enterocins among clinical enterococci strains.

Enterocins 62-6, DD14, FH 99 were all isolated from human samples, including the vaginal tract, meconium of a healthy newborn, and human feces, correspondingly. The first and last referred bacteriocins are encoded on plasmids, whereas the second one is located on the chromosome. The three bacteriocins have relatively narrow antimicrobial activity spectra (Table S1).

Enterocins L50 (EntL50), Q, RJ-11, and 7A/7B were isolated from foodstuffs. EntL50 has been identified either on the chromosome or plasmids of different *E. faecium* strains isolated from fermented foods. This is a broad-spectrum enterocin, composed by EntL50A and EntL50B peptides, that exerts antimicrobial activity against multiple Gram-positive and also Gram-negative bacteria, such as *Escherichia coli, Salmonella enterica, Serratia marcescens*, and *Pseudomonas fluorescens* (Table S1) [79]. Freitas et al. (2016) screened enterocins L50A and L50B in VREfm and VSEfm outbreak isolates without positive results [56]. Enterocin Q and RJ-11 are produced by *E. faecium* L50 isolated from a dry fermented sausage and *E. faecalis* RJ-11 isolated from rice bran, respectively. Both enterocins showed antimicrobial activity against *Enterococcus* species (Table S1) [62,80]. Enterocin 7A/B is produced by *E. faecalis* 710C, isolated from a beef product. It has a broad spectrum of antimicrobial activity, being effective against several Gram-positive bacteria, such as *Carnobacterium* sp., *Clostridium* spp., *Listeria* spp., methicillin-resistant *Staphylococcus aureus*, and VREfm (Table S1). MR10A/B and EJ97 are two bacteriocins produced by *E. faecalis* strains, respectively isolated from bird uropygial glands and municipal wastewater. MR10 is chromosome encoded, whereas EJ97 is encoded on a 60-kb plasmid. Both are effective against species of enterococci, *Bacillus*, *Listeria*, and *Staphylococcus* (Table S1). Finally, K1 is an enterocin about which little is known, produced by *E*. *faecium* and highly potent against *E. faecium*, including VREfm (Table S1).

#### 2.1.3. Class II—Other Bacteriocins

Those bacteriocins that do not share the basic characteristics with the already labeled bacteriocins, or that there is uncertainty on the subclass to which they should belong, were placed together, without classification (Table S1). These bacteriocins have been identified in different enterococci species recovered from human and food samples. Among them, bacteriocin 32 (Bac32), 51 (Bac51), EF478, and enterocin B (EntB) were detected in clinical *E. faecium* and *E. faecalis* isolates (Table S1).

Bac32 was firstly identified in clinical VanA-VREfm and VSEfm isolates in the USA and Japan, and also in a nonclinical *E. faecium* isolated from healthy feces in Japan. Inoue et al. (2006) described that Bac32 genes were located on a 12.5-kb highly transferable plasmid, pTI1-type, which was spread among different VREfm strains according to PFGE patterns [81]. Genes encoding Bac32 were located on a different plasmid than the vancomycin-resistance genes [18], a common finding to that reported by Freitas et al. (2016) describing Bac32 on small plasmids of 10–12 kb [56]. In the latter study including outbreak VREfm from different countries, Bac32 was identified at low rates and in association with common clones of the same region, which suggests clonal and/or regional expansion may influence Bac32-carrying plasmid spread [56]. This enterocin has an antimicrobial spectrum that includes *E. faecium*, *E. durans*, and *E. hirae* strains (Table S1) [81].

Bac51 was also identified in a clinical VanA-type VREfm from Japan and located on a small 6.0-kb mobilizable plasmid, pHY. Bac51 shows antimicrobial activity against *E. faecium*, *E. durans*, and *E. hirae* strains [82].

Although EntB was first isolated from *E. faecium* T136 of a Spanish dry sausage, it has also been found in cheese samples and among clinical isolates [55,56,58]. Freitas et al. (2016) and Strateva et al. (2015) described the presence of EntB as infrequent in VREfm and VSEfm clinical isolates [56,57]. Regarding the first study, the VREfm isolates harboring EntB were mostly from Latin America, thus suggesting the possibility of a regional spread of particular clones (EntB was occasionally located on the chromosome) or plasmids [56]. EntB is often identified in strains co-carrying EntA and they have synergistic effects. It is active against a wide spectrum of Gram-positive bacteria, especially spoilage and foodborne pathogens, such as *Clostridium tyrobutyricum*, *C. sporogenes*, *S. aureus*, and *L. monocytogenes* (Table S1) [59].

Bacteriocin EF478 was obtained from a stool sample of a hospitalized patient in Thailand [36]. The bacteriocin producer, *E. faecalis* E478, showed a potent antimicrobial activity against clinical multidrug-resistant enterococci (MDRE) isolates (not specified in the study), including VRE (Table S1). These characteristics make EF478 a promising antimicrobial candidate as anti-MDRE and VRE, with further studies needed.

Enterocins 96, F4–9 and durancin 61 A were found in *E. faecalis* and *E. durans* species obtained from different types of food [83,84,85]. All three are mostly active against different enterococci species, whereas durancin 61 A showed antimicrobial activity against several pathogens, such as clinical drug-resistant *Clostridiodes difficile*, methicillin-resistant *S. aureus*, and VREfm (Table S1). As for enterocin IT and ESL5, they are produced by *E. faecium* IT62 isolated from Italian ryegrass in Japan and *E. faecalis* SL-5 isolated from healthy human feces, correspondingly [86,87]. Enterocin IT is active mostly against *Enterococcus* species, whereas ESL5 showed antimicrobial activity against *Bacillus cereus, Bacillus subtilis, L. monocytogenes, Cutibacterium acnes*, and *S. aureus.* (Table S1).

#### 2.1.4. Class III—Bacteriolysins

Bacteriolysins are large-molecular-weight (>10 kDa) lytic enzymes that degrade the cell walls of target bacteria [18]. To date, there are two known enterococcal bacteriolysins, enterolysin A and bacteriocin 41 (Bac41), both described in *E. faecalis* (Table S1).

Enterolysin A was described in *E. faecalis* LMG 2333 isolated from Iceland fish, in *E. faecalis* DPC5280 extracted from an Irish raw milk sample, and also in an *E. faecalis* strain isolated from artisanal cheeses [57,88,89]. It has a broad spectrum of antimicrobial activity against Gram-positive bacteria, namely *E. faecalis*, *L. innocua*, *L. lactis* strains, among others (Table S1).

Bac41 was isolated from a clinical *E. faecalis* YI714 strain on a 61-kb conjugative pheromone-responsive plasmid, pYI14, and seems to have a narrow spectrum of antimicrobial activity, being only active against *E. faecalis* (Table S1). Bac41-like genes were additionally identified in outbreak vancomycin-resistant *E. faecalis* strains in Japan as co-located with *vanB* on a pheromone-responsive large conjugative plasmid pMG2200 (about 106 kb), demonstrating the acquisition of different adaptive traits on the same mobile genetic element [90]. Interestingly, Bac41 was described as disseminated in *E. faecalis* clinical strains presenting partial diversity in the region downstream the *bac* gene linked to specific immunity factors for self-resistance [91]. The authors suggested that these Bac41 subtypes may have arisen to adapt immunity specificity as an advantage for competition between strains, and that Bac41 can be more efficient than toxin–antitoxin systems in maintaining population levels.

## 3. Diversity of Enterocins in Clinical Enterococci

The production of bacteriocins by commensal bacteria can modulate niche competition between enterococci and gut competitors, aiding in the establishment of a stable niche and promoting a healthy microbiota. At the same time, bacteriocins are common among clinical enterococci isolates and their bacteriocinome may have a greater role in their invasion during infection than currently recognized [92]. Hospitalized patients often show gut dysbiosis scenarios, with enterococci expansion described, especially in long-stay patients under antibiotics therapy [93]. Several enterococci features have been suggested to contribute to this scenario as antibiotic resistance, ability to use particular carbohydrates, diverse virulence factors, and a plethora of mobile genetic elements [94], with the role of enterocins in these dynamics greatly underexplored.

Most known enterocins are widespread in isolates from human and nonhuman sources, and a few seem to be particularly associated with clinical enterococci (Table S1) [18]. Different reasons may account for these observations, including the fact that screening and characterization of enterocins have been greatly made in food/dairy samples [18] and, in general, poor attention has been given to enterocin genes regarding clinical/outbreak strains. In fact, we lack sufficient robust data about the distribution of enterocins among enterococci from different hosts and habitats for the establishment of a stronger niche association. Moreover, data analysis must be careful, since it could be biased by the fact that not all types of enterocins are equally searched in available studies. Data are strongly dependent on available bacteriocin knowledge in a particular period, namely, of their distribution in particular niches, species, or local distribution of transferable genetic elements, as well as study design. Here we will summarize epidemiological studies assessing the presence of enterocins in clinical enterococci strains.

According to some of the first epidemiological available studies, the percentage of enterocin-producing isolates appears to be higher amongst clinical samples than among fecal or environmental samples [17,35,64,80]. Del Campo et al. (2000) found that 63% of human clinical isolates were enterocin producers, contrasting with <40% of enterococci from other origins [65]. Likewise, Phumisantiphong et al. (2017) reported a similar tendency, in which 49% of clinical isolates were enterocin producers, followed by environmental (10%) and water isolates (0.82%) [36]. Cytolysin has also been greatly associated with clinical *E. faecalis*, although not exclusively, being associated with a hemolytic phenotype, and a higher virulence in animal models [45]. Different studies also confirmed a higher occurrence of Bac32 and Bac43 among clinical VREfm/VSEfm than among nonclinical *E. faecium* isolates, with Bac43 being exclusively found in clinical VREfm [55,63,80]. A study performing a detailed location of plasmid and bacteriocin genes in clonally diverse outbreak VREfm from different countries and clinical VSEfm from Spain described a high prevalence of EntA, but Bac32, Bac43, EntB, and EntP rates were variable and detected at lower rates [56]. Genes coding for Bac32 or Bac43 were consistently located on small theta-replicating plasmids of 12–18 kb, while the gene encoding enterocin P was linked to large plasmids of ca. 150–200 kb, but these plasmids were not carriers of vancomycin resistance genes. As previously mentioned, the bacteriocins searched were limited, most probably skewing results.

More recently, the increasing number of genomic-associated studies are shedding light on the relevance that enterocins might have in the adaptation of enterococci into the hospital environment [29,94,95]. Raven et al. (2016) identified an uncharacterized enterocin-encoding gene among 33 out of 34 clonally diverse *vanB*-positive *E. faecium* that was not present in VanA-positive isolates [95]. The authors stated that the presence of this bacteriocin may add a fitness benefit, explaining the 5-year persistence of phenotypically vancomycin-susceptible isolates carrying chromosomal *vanB*-transposons. Zhou et al. (2018) identified a bacteriocin co-located with chromosomal *vanB*::Tn*1549* transposons in different outbreak VREFm strains [96]. The co-location of a bacteriocin with *vanB* transposons has also been described in the case of Bac41, which was associated with the pheromone-responsive plasmid pMG2200 in outbreak *E. faecalis* strains [90]. The nature of the former bacteriocin is lacking, but both studies illustrate the co-location of bacteriocin and vancomycin resistance on the same genetic element. Zheng et al. (2009) additionally showed outbreak *E. faecalis* strains co-carrying different pheromone-responsive plasmids, pMG2200 encoding VanB-type vancomycin resistance and Bac41, and pMG2201 encoding erythromycin resistance and cytolysin, thus showing that the same strain can acquire different mobile elements, providing a selective advantage in the clinical setting [90]. Moreover, the bacteriocin system may serve as a toxin–antitoxin system in order to increase plasmid stability and, consequently, prevent the loss of the plasmid-encoding antibiotic-resistance genes. These different examples convey the same message that these enterocins should play a relevant role in the maintenance and spread of such vancomycin-resistant genetic elements.

Other wide genomic studies include that of Arredondo et al. (2020), who analyzed the genomes of 1644 diverse *E. faecium* isolates and showed that specific genes, including bacteriocin ones, were exclusively present in plasmidome populations from hospitalized patients [97]. In common to previous suggestions [91], the authors mentioned that this hypothetical bacteriocin can act as a toxin–antitoxin system in which cells that do not bear the bacteriocin plasmid are excluded. Pöntinen et al. (2021) also scrutinized over 2000 *E. faecalis* genomes and found that already, in 1962, a clinical strain carried a bacteriocin on an 80 kb plasmid, along with a toxin–antitoxin system and metal resistance genes [30].

Some enterocins have been essentially identified in clinical isolates: Bac32, Bac43, Bac51, and RC714 among *E. faecium*, and Bac21, Bac41, and EF748 among *E. faecalis* strains (Table S1). Only Bac32 and Bac43 were further identified in one healthy individual each [64,81]. Notably, these enterocins were located on conjugative pheromone-responsive plasmids among *E. faecalis* and on small mobilizable plasmids among *E. faecium*. Both plasmid types are part of the plasmidome commonly associated with each species, either co-carrying other adaptive traits (e.g., antibiotic resistance on pheromone-like plasmids) or not. Small mobilizable plasmids disseminated in clinical *E. faecium* strains seem to be usually cryptic (without known function), but frequently carry bacteriocin genes [56,77]. This, together with the fact that clinical strains present a plasmidome highly dissimilar to that of other hosts [56,97], stresses a role for plasmids or other mobile genetic elements in the killing of competing lineages. Whether the production of enterocins can be more attributed to *E. faecalis* or *E. faecium* species is still debatable given the scarcity of wide studies on this subject. Ness et al. (2014) suggested that *E. faecium* may be one of the greatest producers of enterocins among the fecal LAB microbiota [18]. Del Campo et al. (2000) reported that, among clinical isolates, 82% of *E. faecalis* were bacteriocin producers, whereas only 22% *E. faecium* produced those peptides [65]. To better understand such dynamics, enterocin-carrying genetic elements need to be better characterized, as well as their ability to transfer or be mobilized by conjugative elements, not forgetting as well the occurrence of toxin/antitoxin systems on the same elements that, among other factors, can contribute to the species-specificity of enterocins. Complicating the scenario is the fact that the bacteriocin activity spectrum may differ against different strains of the same species, a hypothesis underexplored that may bring relevant answers in the complex context of strain niche control needed for enterococci invasion under antibiotic treatments.

## 4. Use of Bacteriocins to Fight against VRE Human Infections

The expansion of VRE in the human gut has been associated with a higher risk of infection, as well as diversification of clones with variable antimicrobial resistance profiles, which could impair a successful therapy, thus generating higher costs and risk of death [98,99,100]. MDRE have been a global menace for many years and, despite new therapeutic alternatives and hygiene measures, they remain highly transmissible among patients and a problem to solve in many countries [101,102]. In the EU and European Economic Area (EEA), the number of VRE infections and deaths nearly doubled between 2007 and 2015 [98] and ranked as the second greatest burden in terms of disability-adjusted life years (91.1%) [98,100]. In 2019, the percentage of invasive VREfm was between 1% and 5% in Spain, Norway, and Sweden, but, in Germany, Poland, and Croatia, it was between 25% and 50% [103]. According to the 2019 CDC report, VRE infections caused, in the USA, 54,500 hospitalizations and 5400 deaths, with USD 539 million in healthcare costs in 2017 [104]. Given the dramatic scenario of managing VRE infections, which usually are caused by strains resistant to multiple antibiotics besides vancomycin, no viable options remain, with bacteriocins being a potential adjuvant in the treatment of severe VRE infections.

The number of studies assessing the bacteriocinogenic activity against VRE is limited, but increasingly growing along with the recent boom of microbiome research studies [13]. Farias et al. (2021) tested the potency of EntP against 14 VanA *E. faecium* and *E. faecalis* and concluded that all the VRE isolates were sensitive to the bacteriocin [105]. Similarly, Phumisantiphon et al. (2017) tested the antimicrobial activity of bacteriocin EF478, produced by *E. faecalis* 478, against 68 MDRE and VRE clinical isolates, and found that EF478 was able to inhibit 41% of them [36]. Lastly, Fugaban et al. (2021) reported that *E. faecium* ST651ea, carrying EntB and EntP genes, and *E. faecium* ST7119ea and ST7319ea, harboring EntA and EntB genes, displayed strong antimicrobial activity against most clinical VRE isolates tested [106]. Focusing on non-enterococcal bacteriocins, Severina et al. (1998) demonstrated that nisin was effective in reducing viable cells of VRE, whereas Piper (2009) showed that lacticin 3147, produced by *Lactococcus lactis* was highly potent against VREfm and VREfs [107,108]. Pumicilin 4, a bacteriocin produced by a *Bacillus pumilus* strain, also showed antibacterial activity against VREfs [109]. Other examples of enterocins and other bacteriocins presenting activity against VRE are described in the literature [110].

Although these data represent promising alternatives to control VRE expansion in a patient’s gut or to treat VRE infections, such good activity needs to be confirmed by in vivo models, as many factors (e.g., bacteriocin producers’ survival and density, microbiota interaction, quorum sensing events) might determine the success of bacteriocin therapy [13]. Millette et al. (2008) were the first to demonstrate that both nisin-Z- and pediocin PA-1-producing strains are capable of not only modulating the gut microbiota, but also of reducing the intestinal colonization of VRE in a mouse model. This pair of bacteriocins, isolated from *L. lactis* MM19 and *Pediococcus acidilactici* MM33, correspondingly, showed a great potential as antimicrobials able to control intestinal infections by VRE in vivo [111]. Additionally, the lantibiotic NAI-107 was found to be effective against VRE in vitro and Jabés et al. (2011) was able to prove this result in vivo. NAI-107 was administrated intravenously to neutropenic mice and was highly potent against VanA *E. faecium* 569 and VanA *E. faecalis* A533 [112]. More recently, Kim et al. (2019) showed that a four-strained consortium of commensal bacteria (*Clostridium bolteae*, *Blautia producta*, *Bacteroides sartorii*, and *Parabacteroides distasonis*) restored colonization resistance against VREfm in antibiotic-treated mice [113]. Then, they showed that *Blautia producta* BP_SCSK_ reduced VREfm growth through the secretion of a lantibiotic similar to the nisin-A produced by *L. lactis* [113]. Although VRE growth is inhibited by *B. producta* BP_SCSK_ and by *L. lactis* in vitro, only *B. producta* BP_SCSK_ colonized the colon and decreased VRE density in vivo, with reduced activity against other intestinal commensal bacteria. These authors also demonstrated a direct correlation between the amount of the bacteriocin gene and VRE reduction in germ-free mice containing patient feces [113]. There are a few studies addressing the effects of probiotic strains on VRE clearance of the gut microbiome; however, the specific mention to the bacteriocins they may carry acting as the driving force is lacking. For instance, Ubeda et al. (2013) reported that obligate anaerobic bacteria from the *Barnesiella* genus are able to clear VRE from the intestinal microbiota of mice [114]. Moreover, in two randomized controlled trials, one addressing adult nephrology patients and the other focusing on hospitalized pediatric patients, the authors reported that the ingestion of *Lactobacillus rhamnosus* GG temporarily eliminates the gastrointestinal carriage of VRE [115]. Although these were favorable outcomes, there is the need to address the safety of the probiotic strain in immunocompromised patients, since that is still not clear. Moreover, it is important to understand if there was an absolute clearance of VRE of the organism or whether the numbers of VRE were under the level of detection.

Despite the aforementioned constraints, a handful of molecules showing bacteriocinogenic activity against VRE are already in preclinical stages. For example, microbisporicin NAI-107 (Naicons SRL and Sentinella Pharmaceuticals), a mersacidin analog (Novacta Biosystems Ltd., Welwyn Garden City, UK), and AS-48 have proven to be clinically important by showing antimicrobial activity against relevant pathogens, as VRE [116]. Although most of these are associated with in vitro findings, these bacteriocins appear to be promising candidates as alternatives to antibiotics. Despite the insufficient investment and the study of peptides that have not been bioengineered and thoroughly optimized, future research may bring innovative strain-specific approaches into the identification of highly active narrow-spectrum bacteriocins targeting relevant pathogens as VRE, either alone or as antibiotic adjuvants [117].

## 5. Challenges of Bacteriocin Use

**Figure 2:**
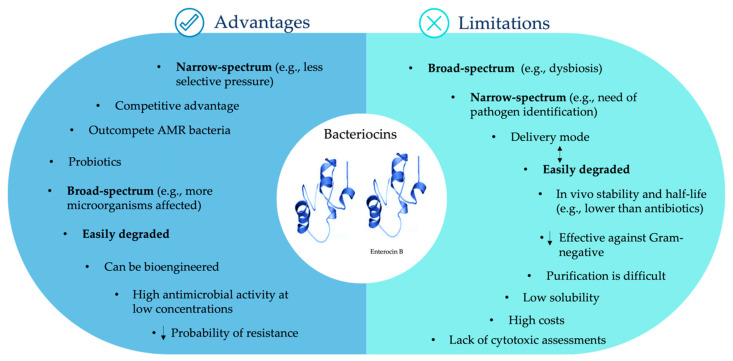
Advantages and limitations of bacteriocins. Words in bold indicate that such a bacteriocin feature can be an advantage or a limitation, according to different contexts. Arrows pointing down mean “less”. Arrows pointing simultaneously up and down mean “are related”.

As mentioned before, bacteriocins provide a variety of advantages to the gut microbiota (Figure 2). Further advantages include their easy degradation by proteases in the gastrointestinal tract, which makes them safe for human and animal consumption [118,119]; their gene-encoded nature, which enables them to be bioengineered, increasing their potency or specificity against target bacteria [25,43]; and their high antimicrobial activity at low concentrations, often in the nanomolar range (Figure 2) [11].

Regardless of the plethora of advantages that bacteriocins can offer, they still present some limitations, especially because of few in vivo studies, as the data from these could differ from in vitro studies due to constraints associated with probiotic strain survival, bacteriocin bioavailability and degradation, the influence of the gut microbiota in bacteriocin production, among others (Figure 2). Here, we described some of the current challenges associated with the possible bacteriocin use as therapeutic agents for bacterial infections, namely caused by enterococci.

Although broad-spectrum bacteriocins have been an attraction for food preservation due to their wide antimicrobial activity and expected safety, some studies already reported that, if they would be used as therapeutic agents, they could induce an unbalance among microbiota phyla (e.g., increase of Proteobacteria at the expense of Firmicutes and Bacteroidetes) similar to the broad-spectrum antibiotics [25,120]. On the other hand, the narrow spectrum of several bacteriocins, an advantage for microbiota maintenance, can be a limitation if we think that the causal strain of an infection needs to be accurately identified prior to bacteriocin treatment. Fortunately, recent strain-typing developments (mainly by genomics, but also spectroscopic assays) may evolve towards a routinely fast and cost-effective strain identification [121,122].

A major drawback among antimicrobial compounds is bacterial resistance. Bacteriocin resistance seems to be less probable to happen when compared to antibiotics, with several factors contributing to that, namely: (i) bacteriocins of narrow spectrum only target specific bacteria, reducing the selective pressure on the nontargeted ones [10]; (ii) they can have different mechanisms of action (those who target the cell envelope and those that act within the cell, disturbing the expression of genes and protein production) [10]; (iii) they normally act fast, which decreases the possibility to develop resistance, if only susceptible cells are present [41]; (iv) bacteriocins interact with cell receptors that are so far known as different from those used by antibiotics, making cross-resistance among both types of antimicrobials less plausible [123]. Nevertheless, different bacterial mechanisms have been described to confer resistance to bacteriocins, which could be innate or acquired. They include immunity mimicry (functional homologues of bacteriocin immunity systems), bacteriocin degradation by bacterial enzymes, changes in bacterial cell wall and membrane, or mutations in regulatory elements [124]. Among enterococci, Drapper et al. (2009) reported that *E. faecium* DO strains were resistant to lacticin 3147 through immunity mimicry [125]. Another study described that *E. faecalis* strains can degrade and inactivate pediocin-like bacteriocins, which seemed to be related to the production of gelatinase, a metalloendopeptidase [126]. A number of studies have reported that bacteriocin resistance in class IIa bacteriocins involves downregulation of Man-PTS expression in both natural resistant isolates and spontaneous resistant mutants [42]. As an example, mutants of *E. faecalis* V583 resistant to pediocin PA-1 were found to have lower expression of the *mpt* operon encoding the mannose-specific phosphoenolpyruvate carbohydrate phosphotransferase system (PTS) in comparison to the wild-type strain [127]. Studies using mutants of *E. faecalis* and *E. faecium* resistant to pediocin PA-1 and mundticin KS, respectively, reported that changes in the membrane charge and composition were related to bacteriocin resistance [128,129]. Recently, spontaneous *E. faecalis* mutants showed resistance to the antimicrobial effects of BacL1 and BacA (from the bacteriolysin Bac41) due to a truncation deletion on the GalU protein leading to a cell-wall-associated polysaccharide defect [130]. Cases of cross-resistance among bacteriocins have also been reported. For example, nisin resistance in *E. faecium* DSMZ 20477 and in *E. faecalis* ATCC 29212 was found to confer cross-resistance to enterocin FH99 and pediocin 34 [131]. Knowledge of how bacteriocin resistance emerges and may evolve in vivo, namely among enterococci, is rather incomplete, with further investigations needed. A potential approach to avoid bacteriocin-resistant bacteria could be the combination of bacteriocins with different mechanisms of action or even combination of bacteriocins with other antimicrobial agents, such as conventional antibiotics, thus broadening their spectrum of action and antimicrobial activity [43]. Besides preventing resistance, combining bacteriocins with antibiotics can also have a lower impact on the intestinal microbiota. By working synergically, the dosage of both drugs can be lowered; hence, the side effects inherent to antibiotic treatment would be, in theory, slighter [11].

Bacteriocin challenges also include their delivery mode, which needs to protect bacteriocins from the degradation activity of enzymes produced by animals and humans (e.g., of the upper digestive tract and the stomach, when peptides are administered orally) that could affect their bioavailability and efficacy [132]. They are expected to have lower in vivo stability and half-life than antibiotics [133]. On one hand, being easily degraded makes bacteriocins safe for human and animal consumption. To overcome limitations, such as the degradation by proteolytic enzymes, nano-encapsulation may be the answer [10]. Furthermore, bioengineering strategies can be employed to manipulate the peptides, making them nonrecognizable by proteases and even enhancing other qualities, such as potency and effectiveness against Gram-negative bacteria, which are usually more resistant to bacteriocin activity due to their outer membrane [10,43].

Another disadvantage is the bacteriocins’ complex nature, making purification a difficult process and the costs of production highly elevated, so their synthesis is impractical for large-scale production [116], making it necessary to come up with more suitable strategies, such as simplifying the purification protocols. Moreover, the long and/or hyper-hydrophobic peptide hampers bacteriocin solubility and promotes self-aggregates [133]. Even though the investment in the development of bacteriocins for clinical applications is not significant, given the difficulties of large-scale production, some innovative modes of generating improved variants (e.g., cell engineering, chemical synthesis, use of a defined media, designing strains, among others) are under clinical development [134].

Finally, the lack of cytotoxic assessments, which are key factors precluding the exploitation of bacteriocins for clinical applications, namely as antimicrobial therapeutic agents, has also been a challenge for the development and application of bacteriocins [116]. For all the reasons described, the clinical use of bacteriocins is highly dependent on future bioengineering, large-scale production developments, besides further studies about pharmacokinetic, pharmacodynamics, and toxicity features.

## 6. Future Perspectives

Bacteriocins are powerful antimicrobial peptides naturally synthesized by certain bacteria that represent a potential solution to combat the AMR crisis given their abundance and diversity. Currently considered by some authors as the future antibiotics [10,43], they are a promising alternative to combat MDR infections, namely caused by VRE, a global priority pathogen and a major public health issue in many regions. Understanding the dual role of bacteriocins as modulators of gut microbiota, as well as potential therapeutic alternatives is critical to potentiate them as a solution to control and treat such superbugs.

Enterocins can be a great promise to fight the antimicrobial resistance crisis, alone or together with other antimicrobial or preventive strategies (e.g., antibiotics, bacteriophages, vaccines). However, similarly to bacteriocins produced by other bacteria, a greater investment and more studies need to be conducted to position them as marketable therapeutic agents, namely related to cytotoxicity, immunogenicity, delivery systems, or development of enterocin resistance. Although studies on enterocins started many decades ago, they have several limitations, including that activity-screening approaches target only one strain of each species in most cases, enterocin genes are identified without evaluating antimicrobial activity or they do not take into account the interaction with competing strains/species. Nevertheless, the current and increasing wide use of genomic, metagenomic, and immunological strategies will surely impulse the identification of new enterocin biosynthetic gene clusters and deepen our knowledge about their features in the face of the complex network of gut microbiota and the immune system of different hosts, enabling to classify them as safe to be used as probiotics or postbiotics. Indeed, an exponential number of studies make use of public and user-friendly databases (e.g., antiSMASH, BACTIBASE, BAGEL, LABiocin, and more) that facilitate the in silico mining of genomes containing AMPs and bacteriocins, mostly derived from Gram-positive bacteria [135,136,137,138]. These web-accessible databases are getting more and more specialized (for example LABiocin is a specialized database on LAB bacteriocins) and unveil an exponentially growing field with the number of bacterial genomes that are released daily. On the other hand, genomic and metagenomic approaches can further provide data about the variability of bacteriocinomes in different enterococcal populations and the role of enterocins in the expansion of MDR enterococci and mobile genetic elements, including at the strain level, in hospitalized patients’ gut, with consequent invasion and infection. Certain that their full biological role in nature is yet to be discovered, the combination of robust in vitro and in vivo studies within different microbial community contexts, together with genome mining and modern industrial processes of bacteriocin production, is warranted to ensure the application of enterocins in relevant clinical and biomedical contexts.

## Supplementary Materials

The following are available online at https://www.mdpi.com/article/10.3390/antibiotics10101215/s1, Table S1: Classification, source of isolation, activity spectrum and localization of known enterocins.

## Abbreviations

AMPs	Antimicrobial peptides
AMR	Antimicrobial resistance
Bac	Bacteriocin
ECDC	European Centre for Disease Prevention and Control
EEA	European Economic Area
EFSA	European Food Safety Authority
EMA	European Medicines Agency
Ent	Enterocin
EU	European Union
FDA	Food and Drug Administration
GRAS	Generally recognized as safe
LAB	Lactic Acid Bacteria
Man-PTS	Mannose phosphotransferase system
MDRE	Multidrug-resistant enterococci
PTS	Phosphoenolpyruvate Carbohydrate Phosphotransferase System
QPS	Qualified Presumption of Safety
QS	Quorum sensing
US	United States
VRE	Vancomycin-resistant enterococci
VREfm	Vancomycin-resistant *E. faecium*
VREfs	Vancomycin-resistant *E. faecalis*
VSE	Vancomycin-susceptible enterococci
VSEfm	Vancomycin-susceptible *E. faecium*
WHO	World Health Organization
