# Supplementary material for: Enterococcus spp. as a Producer and Target of Bacteriocins: A Double-Edged Sword in the Antimicrobial Resistance Crisis Context
Source: Antibiotics (Basel). 2021 Oct 7;10(10):1215. doi: 10.3390/antibiotics10101215 (PMC8532689; doi:10.3390/antibiotics10101215)
Supplement: Supplementary file 1 [file antibiotics-10-01215-s001.zip › antibiotics-1357584-supplementary.pdf]

Table S1. Classification, source of isolation, activity spectrum and localization of known enterocins.

Enterocins classes	*Enterococcus* spp. producer strain	Source/year of isolation	Activity spectrum	Localization	References
**Class I: Lantibiotics (<5 kDa)**					
Cytolysin (hemolysin)	*E. faecalis*	Clinical isolates<br>Food<br>Animals<br>Healthy infants<br>(1983- 1987)	*Enterococcus* spp.<br>LAB strains<br>Eukaryotic cells	Pheromone-responsive plasmid pAD1 (59.6 kb)<br>or chromosome	[1]
	E. faecalis	VanB-*E. faecalis* clinical isolates	*Enterococcus faecalis*	Plasmid pMG2200 (106 kb)	[2]
Enterocin W (Wα and Wβ)	*E. faecalis* NKR-4-1	Thai fermented fish	*Bacillus coagulans*<br>*Bacillus circulans*<br>*Listeria innocua*<br>*Pediococcus pentosaceus*<br>*Enterococcus faecalis*<br>*Lactococcus lactis*<br>*Lactobacillus sakei*	Unknown	[3]
**Class II: Non-lantibiotics (<10 kDa)**					
**<u>Class IIa: Pediocin-like bacteriocins</u>**					
Enterocin A	*E. faecium* CTC492<br>*E. faecium* P21<br>*E. faecium* T136	Spanish dry fermented sausage	*Lactobacillus sake*<br>*Pediococcus acidilactici*<br>*Pediococcus pentosaceus*	Chromosome<br>Unknown<br>Chromosome	[4]<br>[5]<br>[6]
	E. faecium BFE 900	Black olives	*Lactobacillus plantarum*<br>*Listeria innocua*	Unknown	[7]
	E. faecium EFM01<br>*E. faecium* DPC1146<br>*E. faecium* WHE 81<br>*E. durans* BGGO9-30<br>*E. faecium*	Dairy sources	*Listeria monocytogenes*<br>*Enterococcus faecalis*<br>*Enterococcus faecium*<br>Bacteriocinogenic activity not assessed<br>Bacteriocinogenic activity not assessed	Unknown<br>Chromosome<br>Chromosome<br>Unknown<br>Unknown	[8]<br>[9]<br>[10]<br>[11]<br>[12]

		Artisanal cheeses from Bulgaria			
	E. faecium N15	Japanese rice-bran paste		Unknown	[13]
	E. faecium	Clinical VREfm and VSEfm isolates (since 1991)	Bacteriocinogenic activity not assessed	Chromosome	[14]
	E. faecium	Clinical isolates from Bulgaria (2012-2015)	Bacteriocinogenic activity not assessed	Unknown	[12]
Enterocin P	*E. faecium* P13<br>*E. faecium* L50<br>*E. faecium* AA13<br>*E. faecium* G16	Spanish dry fermented sausages	*Listeria monocytogenes*<br>*Clostridium perfringens*<br>*Clostridium botulinum*<br>*Clostridium sporogenes*<br>*Staphylococcus carnosus*<br>*Staphylococcus aureus*<br>*Enterococcus faecalis*<br>*Propionibacterium* species	aChromosome	[15–17]
	E. faecium 86	Meat pie	VanA *E.faecium* strains<br>VanA *E. faecalis* strains<br>*Listeria monocytogenes*	Plasmid	[18]
	E. faecium	Artisanal cheeses from Bulgaria	Bacteriocinogenic activity not assessed	Unknown	[12]
	E. faecium	Clinical isolates from Bulgaria (2012-2015)	Bacteriocinogenic activity not assessed	Unknown	[12]
	E. faecium	Clinical VREfm and VSEfm isolates (since 1997)	Bacteriocinogenic activity not assessed	Plasmid	[14]
Bacteriocin 43	*E. faecium* VRE82	VREfm clinical isolates (1994-1999)<br><br>Healthy Japanese student (2002-2003)	*Enterococcus faecalis*<br>*Enterococcus faecium*<br>*Enterococcus hirae*<br>*Enterococcus durans*<br>*Listeria monocytogenes*	Plasmid pDT1-like (6.2 kb)	[19]

	E. faecium	Clinical VREfm and VSEfm isolates (since 1998)	Bacteriocinogenic activity not assessed	Small theta-replicating plasmids (ca. 7 kb)	[14]
Bacteriocin RC714	*E. faecium* RC714	Clinical sample (Human exudate) (1996)	*Listeria* spp.<br>VanA and VanB *Enterococcus faecalis*<br>VanA *Enterococcus faecium*<br>*Lactobacillus paracasei*<br>*Lactobacillus plantarum*<br>*Leuconostoc spp.*<br>*Pediococcus pentosaceus*	Unknown	[20]
Bacteriocin 31	*E. faecalis* YI717	Clinical isolate	*E. hirae* 9770<br>*Listeria monocytogenes*<br>*Enterococcus faecium*	Pheromone-responsive conjugative plasmid pYI17 (57.5 kb)	[21]
	E. durans strains	Dairy sources	Bacteriocinogenic activity not assessed	Unknown	[11]
Bacteriocin GM-1	*E. faecium* GM-1	Newborn feces	*Bacillus subtilis*<br>*Bacillus megaterium*<br>*Staphylococcus aureus*<br>*Listeria monocytogenes*<br>*Lactobacillus acidophilus*<br>*Streptococcus thermophilus*<br>*Escherichia coli*<br>*Proteus mirabilis*<br>*Vibrio vulnificus*<br>*Vibrio parahaemolyticus*<br>*Vibrio alginolyticus*<br>*Klebsiella pneumoniae*<br>*Salmonella* Typhimurium<br>*Pseudomonas aeruginosa*	Unknown	[22]
Avicin A	*E. avium* XA83<br>*E. avium* 208	Healthy infant feces (2005 and 2007)	*Listeria* spp.<br>*Enterococcus* spp.<br>*Lactobacillus* spp.<br>*Leuconostoc* spp.<br>*Pediococcus* spp.<br>*Carnobacterium* spp.	Chromosome (7 kb DNA fragment)	[23]

Bacteriocin T8	*E. faecium* T8	Children infected with HIV	*Enterococcus faecalis*<br>*Enterococcus faecium*<br>*Enterococcus gallinarum*<br>*Enterococcus hirae*<br>*Listeria monocytogenes*	Plasmid T8 (7 kb)	[24]
Enterocin MC4-1	*E. faecalis* MC4	Root canals of monkeys *(Macaca fasdculaiis)*	*Enterococcus faecalis*<br>*Enterococcus faecium*<br>*Enterococcus gallinarum*<br>*Enterococcus hirae*<br>*Listeria monocytogenes*	Pheromone-responsive, conjugative plasmid pAMS1 (130 kb)	[25–27]
Hiracin JM79	*E. hirae* DCH5	Wild Mallard ducks	*Clostridium botulinum*<br>*Listeria monocytogenes*<br>*Staphylococcus aureus*	Chromosome	[28,29]
Enterocin S37	*E. faecalis* S37	Poultry feces	*Enterococcus faecalis* JH2-2<br>*Listeria monocytogenes* EGDe 107776<br>*Listeria innocua*<br>*Lactobacillus brevis* F1.114<br>*Lactobacillus brevis* F145	Unknown	[30]
Bacteriocin E 50–52	*E. faecium* 50–52	Commercial Russian broiler chicken cecum	*Campylobacter jejuni*<br>*Yersinia enterocolitica*<br>*Yersinia pseudotuberculosis*<br>*Staphylococcus aureus*<br>*Staphylococcus epidermidis*<br>*Listeria monocytogenes*	Unknown	[31]
Enterocin CRL35	*E. mundtii* CRL35	Argentinean artisanal cheese	*Listeria* spp.<br>*Herpes simplex* viruses	Plasmid (50 kb)	[32]<br>[33]
Mundticin QU 2	*E. mundtii* QU 2	Soybean (Japan)	*Enterococcus* spp.<br>*Lactobacillus* spp.<br>*Leuconostoc* spp.<br>*Pediococcus* spp.<br>*Listeria* spp.	Unknown	[34]
Mundticin ATO6	*E. mundtii* ATO6	Chicory endive	*Listeria monocytogenes*<br>*Clostridium botulinum*<br>*Lactobacillus salivarius*	Plasmid pEM6A	[34,35]

			Listeria sake<br>*Leuconostoc paramesenteroides*<br>*Leuconostoc mesenteroides*<br>*Carnobacterium piscicola*<br>*Pediococcus dextrinicus*<br>*Pediococcus pentosaseus*<br>*Enterococcus faecalis*<br>*Enterococcus hirae*<br>*Listeria inócua*<br>*Clostridium botulinum* spores		
Durancin GL	*E. durans* 41D	Mexican-style artisanal cheese	*Listeria* spp.	Plasmid pDGL (8.3 kb)	[36,37]
Mundticin KS	*E. mndtii* NFRI 7393	Grass silage in Thailand	*Listeria monocytogenes*<br>*Enterococcus faecium* IFO1371<br>*Enterococcus faecalis* IFO12964<br>*Enterococcus mundtii* JCM 8731T	Plasmid pML1 (50 kb)	[38]
Enterocin SE-K4	*E. faecalis*	Grass silage in Thailand	*Listeria* spp.<br>*Bacillus Subtilis*<br>*Clostridium beijerinckii*	Plasmid pEK4S (60 kb)	[39]
Enterocin M	*E. faecium* AL41	Sewage sludge	*Enterococcus faecium*<br>*Enterococcus faecalis*<br>*Enterococcus avium*<br>*Staphylococcus aureus*<br>*Listeria innocua*<br>*Listeria monocytogenes*<br>*Escherichia coli*	Unknown	[40]
**Class IIb: Two-peptide bacteriocins**					
Enterocin C	*E. faecalis* C901	Human colostrum	*Actinomyces neuii*<br>*Enterococcus faecalis*<br>*Enterococcus faecium*<br>*Facklamia hominis*<br>*Lactococcus lactis*<br>*Lactobacillus paracasei*<br>*Leuconostoc mesenteroides*	Plasmid pENTC (9 kb)	[41,42]

			Propionibacterium acnes<br>*Staphylococcus caprae*<br>*Staphylococcus epidermidis*<br>*Staphylococcus anginosus*<br>*Staphylococcus untermedius*		
Enterocin 1071 A and B	*E. faecalis* BFE 1071	Mini-pigs feces	*Enterococcus* spp.<br>*Lactobacillus*<br>*salivarius subsp. salivarius*<br>*Listeria innocua*<br>*Micrococcus* sp.<br>*Peptostreptococcus aerogenes*<br>*Propionibacterium*<br>*Freudenreichii subsp*<br>*shermani*<br>*Streptococcus agalactiae*	Plasmid pEF1071 (50 kb)	[43]
	E. faecalis FAIR-E 309	Argentinian cheese	Bacteriocinogenic activity not assessed	Unknown	[44]
	E. durans BGGO8-25<br>*E. durans* BGGO8-26	Dairy sources	Bacteriocinogenic activity not assessed	Unknown	[11]
Enterocin X (Xα and Xβ)	*E. faecium* KU-B5	Sugar apples from Thailand	*Lactococcus lactis*<br>*Enterococcus faecium*<br>*Bacillus circulans*<br>*Bacillus coagulans*<br>*Bacillus subtilis*	Unknown	[45]
**Class IIc: circular bacteriocins**					
Bacteriocin AS-48	*E. faecalis* S-48	Clinical isolates (Human wound exudate)	*Bacillus* spp.<br>*Enterococcus* spp.<br>*Corynebacterium glutamicum*<br>*Corynebacterium bovis*<br>*Nocardia corrallina*<br>*Micrococcus lysodeikticus* LA1000<br>*Pseudomonas fluorescens* CECT385<br>*Pseudomonas aeruginosa* CECTl10<br>*Pseudomona reptilivora*<br>*Enterobacter cloacae*	Conjugative plasmid pMB2 (56 kb)	[46–48]

Bacteriocin 21	*E. faecalis*	Clinical isolate	*Enterococcus faecalis*<br>*Enterococcus hirae* 9790<br>*Enterococcus faecium*<br>*Streptococcus agalactiae*<br>*Streptococcus sanguis*<br>*Streptococcus aureus*	Pheromone-responsive conjugative plasmid pPD1 (59 kb)	[49]
Enterocin 4	*E. faecalis* INIA 4	Raw ewe's milk	*Clostridium tyrobutyricum*<br>*Lactobacillus buchneri*<br>*Lactobacillus brevis*<br>*Enterococcus faecalis*<br>*Enterococcus faecium*<br>*Listeria monocytogenes*<br>*Listeria innocua*	Unknown	[50]
**Class IId: Leaderless bacteriocins**					
Enterocin 62-6	*E. faecium* 62-6	Human vagina	*Lactobacillus* spp.<br>*Streptococcus* spp.<br>*Enterococcus* spp.<br>*Corynebacterium* spp.	Plasmid	[51,52]
Enterocin DD14	*E. faecalis* 14	Meconium of a healthy newborn	*Staphylococcus aureus*<br>*Listeria monocytogenes*<br>*Enterococcus faecalis*<br>*Bacillus subtilis*<br>*Clostridium perfringens*	Chromosome	[53]
Enterocin FH 99	*E. faecium* FH 99	Human feces	*Listeria monocytogenes*<br>*Lactococcus* spp.<br>*Lactobacillus* spp.<br>*Streptococcus* spp.<br>*Enterococcus faecalis* spp.	Possibly plasmid	[54]
Enterocin L50A and L50B	*E. faecium* L50	Dry fermented sausage	*Escherichia coli*<br>*Salmonella enterica*<br>*Serratia marcescens*<br>*Pseudomonas fluorescens*	Plasmid pCIZ1 (50 kb)	[55,56]

	E. faecium 6T1a	Spanish-style green olive fermentation	*Lactobacillus fermentum*<br>*Lactococcus lactis*<br>*Pediococcus pentosaceus*<br>*Clostridium* spp.<br>*Bacillus strains*<br>*Enterococcus faecalis,*<br>*Listeria innocua*<br>*Listeria monocytogenes*	Plasmid pEF1 (23 kb)	[57]
	E. faecium F58	Jben (goat's cheese)	*Listeria. monocytogenes*<br>*Listeria innocua*<br>*Lactococcus lactis*<br>*Staphylococcus aureus*<br>*Bacillus cereus*<br>*Bacillus subtilis*<br>*Brochothrix*<br>*Clostridium perfringens*<br>*Clostridium tyrobutyricum*	Plasmid (22 kb)	[58]
	E. faecium B1 (LMG 19827)<br>*E. faecium* B2 (LMG 19828)	Malaysian tempeh	*Enterococcus faecalis*<br>*Enterococcus faecium*<br>*Carnobacterium divergens*<br>*Chryseobacterium piscicola*<br>*Lactobacillus brevis*<br>*L. pentosus*<br>*Paralactobacillus selangorensis*	Chromosome	[59]
Enterocin Q	*E. faecium* L50	Dry fermented sausage	*Lactobacillus sakei*<br>*Enterococcus faecium*	Plasmid pCIZ2 (7.4 kb)	[16,55]
Enterocin RJ-11	*E. faecalis* RJ-11	Rice bran (1999–2000)	*Bacillus subtilis*<br>*Bacillus amyloliquefaciens*<br>*Listeria monocytogenes*<br>*Enterococcus* spp.	Unknown	[60]
Enterocin 7A and 7B	*E. faecalis* 710C	Beef	*Brochothrix campestris*<br>*Brevundimonas diminuta*<br>*Carnobacterium divergens*<br>*Carnobacterium maltaromaticum*	Unknown	[61]

			Clostridium botulinum spores and viable cells<br>*Clostridium butyricum*<br>*Clostridium difficile*<br>*Clostridium perfringens*<br>*Clostridium sporogenes*<br>*Enterococcus faecium* BFE900<br>*Lactobacillus sakei*<br>*Leuconostoc gelidum*<br>*Listeria innocua*<br>*Listeria monocytogenes*<br>*Pediococcus acidilactici*<br>*Staphylococcus aureus*<br>Methicillin-resistant *Staphylococcus aureus*<br>VREfm strains		
MR10A and MR10B	*E. faecalis* MRR 10-3	Bird (*Upupa epops*) uropyal glands (2004)	*Staphylococcus* sp.<br>*Bacillus licheniformis*<br>*Enterococcus faecium* strains<br>*Lactococcus lactis* LM2301<br>*Listeria innocua* 4030<br>*Listeria monocytogenes* 4032<br>*Staphylococcus aureus* 240<br>*Micrococcus luteus* 241<br>*Bacillus cereus* LWL1<br>*Escherichia coli* U-9	Chromosome	[62]
Enterocin EJ97	*E. faecalis* EJ97	Municipal wastewater	*Enterococcus* spp.<br>*Bacillus* spp.<br>*Listeria* spp.<br>*Staphylococcus aureus*	Plasmid pEJ97 (60 kb)	[63,64]
Enterocin K1	*E. faecium*	Unknown	*Enterococcus faecium*<br>VREfm	Unknown	[65]
**<u>Class II: Other bacteriocins</u>**					
Bacteriocin 32	*E. faecium* VRE200	VREfm clinical isolates from USA (1994-1999)	*Enterococcus faecium*<br>*Enterococcus hirae*<br>*Enterococcus durans*	Plasmid pTI1 (12.5 kb)	[66]

	VSEfm	Clinical isolates from Japan (1990-1993)	*Enterococcus faecium*<br>*Enterococcus hirae*<br>*Enterococcus durans*	Plasmid pTI1 (12.5 kb)	[66]
	E. faecium	Healthy students (2002-2003)	Bacteriocinogenic activity not assessed	Plasmid	[66]
	VREfm	Clinical isolates (since 1992)	Bacteriocinogenic activity not assessed	Plasmid	[14]
Bacteriocin 51	*E. faecium* VRE38	VREfm clinical isolate (1994-2002)	*Enterococcus faecium*<br>*Enterococcus hirae*<br>*Enterococcus durans*	Mobile plasmid pHY (6 kb)	[67]
Enterocin B	*E. faecium* T136	Spanish dry fermented sausage	*Clostridium sporogenes*<br>*Clostridium tyrobutyricum*<br>*Propionibacterium* spp.<br>*Listeria innocua*<br>*Listeria monocytogenes*<br>*Staphylococcus aureus*<br>*Staphylococcus carnosus*<br>*Lactobacillus sake* FVM 148	Chromosome	[6]
	E. faecium	Artisanal cheeses from Bulgaria	Bacteriocinogenic activity not assessed	Unknown	[12]
	E. faecium	VREfm/VSEfm clinical isolates	Bacteriocinogenic activity not assessed	Chromosome (when assessed)	[14]
	E. faecium	Clinical isolates from Bulgaria (2012-2015)	Bacteriocinogenic activity not assessed	Unknown	[12]
Bacteriocin EF478	*E. faecalis* 478	Clinical samples (human feces) (2014-2015)	MDREfm<br>MDREfs<br>VREfm<br>VREfs	Unknown	[68]
Enterocin 96	*E. faecalis* WHE 96	Munster Cheese	*Enterococcus* spp.<br>*Lactobacillus* spp.<br>*Bacillus* spp.<br>*Listeria* spp.<br>*Staphylococcus* spp.	Plasmid	[69]

Enterocin F4-9	*E. faecalis* F4-9	Egyptian salted-fermented fish	*Enterococcus faecalis* JCM 5803T<br>*Enterococcus durans* NBRC 100479T<br>*Escherichia coli* JM109	Unknown	[70]
Durancin 61A	*E. durans* 61A	Fermented milk	Clinical drug-resistant *Clostridium difficile*<br>Clinical VREfm<br>Clinical methicillin-resistant *Staphylococcus aureus*	Unknown	[71]
Enterocin IT	*E. faecium* IT62	Italian ryegrass in Japan	*Enterococcus faecium* WHE 81<br>*Enterococcus faecium* LC 25<br>*Enterococcus hirae* CIP 5855<br>*Bacillus subtilis* CIP 7718<br>*Lactococcus lactis* LC 72	Plasmid pTIT1 (1.38 kb)	[56,72]
Enterocin ESL5	*E. faecalis* SL-5	Human feces	*Bacillus cereus* KCTC 3624<br>*Bacillus subtilis* KFRI 179<br>*Listeria monocytogenes*<br>*Propionibacterium acnes* ATCC 29399<br>*Staphylococcus aureus* KCTC 1927	Unknown	[73]
**Class III: Bacteriolysins (>10 KDa)**					
Enterolysin A	*E. faecalis* LMG 2333	Fish from Iceland	*Lactobacillus* spp.<br>*Lactococcus* spp.<br>*Pediococcus* spp.<br>*Enterococcus faecium* CTC492	Unknown	[74]
	E. faecalis DPC5280	Irish raw milk	*Enterococcus faecalis* DPC1146<br>*Lactococcus lactis* HP<br>*Lactococcus lactis* DPC3147<br>*Lactococcus lactis* CNRZ481<br>*Lactococcus lactis* DPC141<br>*Lactobacillus fermenticum* DPC3310<br>*Pediococcus pentasaceus* DPC5063<br>*Listeria innocua* DPC3306	Unknown	[75]
	E. faecalis	Artisanal cheeses from Bulgaria	Bacteriocinogenic activity not assessed	Unknown	[12]
Bacteriocin 41	*E. faecalis* YI714	Clinical isolate	*Enterococcus faecalis*	Conjugative plasmid pYI14 (61 kb)	[76]

	VanB-*E. faecalis* clinical isolates	*Enterococcus faecalis*		Plasmid pMG2200 (106 kb)	[2]

Abbreviations: LAB – Lactic acid bacteria; VRE – vancomycin resistant enterococci; VREfm - vancomycin resistant *E. faecium;* VREfs - vancomycin resistant *E. faecalis;* VSEfm - vancomycin susceptible *E. faecium;* MDREfm – Multidrug-resistant *Enterococcus faecium*; MDREfs – Multidrug-resistant *Enterococcus faecalis.* Enterocins without classification were not included in the table: Enterocin V, produced by *E. faecalis,* is effective against *Candida albicans;* Enterocin CCM 4231, produced by *E. faecium* CCM 4231, recovered from the rumen content of a calf, is effective against *Clostridium perfringens, Listeria monocytogenes* and *Staphylococcus aureus;* Enterocin 416K1 produced by *Enterococcus casseliflavus* IM 416K1, isolated from Italian sausages is highly effective against *Listeria monocytogenes*; Enterocin RM6, isolated from *E. faecalis* OSY-RM6, recovered from raw milk, has antimicrobial activity against several Gram-positive bacteria including *Listeria monocytogenes, Bacillus cereus*, and methicillin-resistant *Staphylococcus aureus* [77–80].

References

1. Gilmore MS, Segarra RA, Booth MC, Bogie CP, Hall LR, Clewell DB. Genetic structure of the Enterococcus faecalis plasmid pAD1-encoded cytolytic toxin system and its relationship to lantibiotic determinants. J Bacteriol. 1994;176(23):7335-44.
2. Zheng B, Tomita H, Inoue T, Ike Y. Isolation of VanB-type Enterococcus faecalis strains from nosocomial infections: first report of the isolation and identification of the pheromone-responsive plasmids pMG2200, Encoding VanB-type vancomycin resistance and a Bac41-type bacteriocin, and pMG2201, encoding erythromycin resistance and cytolysin (Hly/Bac). Antimicrob Agents Chemother. 2009;53(2):735-47.
3. Sawa N, Wilaipun P, Kinoshita S, Zendo T, Leelawatcharamas V, Nakayama J, et al. Isolation and characterization of enterocin W, a novel two-peptide lantibiotic produced by Enterococcus faecalis NKR-4-1. Appl Environ Microbiol. 2012;78(3):900-3.
4. Aymerich T, Holo H, Havarstein LS, Hugas M, Garriga M, Nes IF. Biochemical and genetic characterization of enterocin A from Enterococcus faecium, a new antilisterial bacteriocin in the pediocin family of bacteriocins. Appl Environ Microbiol. 1996;62(5):1676-82.
5. Herranz C, Casaus P, Mukhopadhyay S, Martínez JM, Rodríguez JM, Nes IF, et al. Enterococcus faecium P21: a strain occurring naturally in dry-fermented sausages producing the class II bacteriocins enterocin A and enterocin B. Food Microbiology. 2001;18(2):115-31.
6. Casaus P, Nilsen T, Cintas LM, Nes IF, Hernandez PE, Holo H. Enterocin B, a new bacteriocin from Enterococcus faecium T136 which can act synergistically with enterocin A. Microbiology (Reading). 1997;143 (Pt 7)(7):2287-94.
7. Franz CM, Schillinger U, Holzapfel WH. Production and characterization of enterocin 900, a bacteriocin produced by Enterococcus faecium BFE 900 from black olives. Int J Food Microbiol. 1996;29(2-3):255-70.
8. Ennahar S, Deschamps N. Anti-Listeria effect of enterocin A, produced by cheese-isolated Enterococcus faecium EFM01, relative to other bacteriocins from lactic acid bacteria. J Appl Microbiol. 2000;88(3):449-57.
9. O'Keeffe T, Hill C, Ross RP. Characterization and heterologous expression of the genes encoding enterocin a production, immunity, and regulation in Enterococcus faecium DPC1146. Appl Environ Microbiol. 1999;65(4):1506-15.

10. Ennahar S, Asou Y, Zendo T, Sonomoto K, Ishizaki A. Biochemical and genetic evidence for production of enterocins A and B by Enterococcus faecium WHE 81. Int J Food Microbiol. 2001;70(3):291-301.
11. Popovic N, Dinic M, Tolinacki M, Mihajlovic S, Terzic-Vidojevic A, Bojic S, et al. New Insight into Biofilm Formation Ability, the Presence of Virulence Genes and Probiotic Potential of Enterococcus sp. Dairy Isolates. Front Microbiol. 2018;9:78.
12. Strateva T, Dimov SG, Atanasova D, Petkova V, Savov E, Mitov I. Molecular genetic study of potentially bacteriocinogenic clinical and dairy Enterococcus spp. isolates from Bulgaria. Annals of Microbiology. 2015;66(1):381-7.
13. Losteinkit C, Uchiyama K, Ochi S, Takaoka T, Nagahisa K, Shioya S. Characterization of bacteriocin N15 produced by Enterococcus faecium N15 and cloning of the related genes. J Biosci Bioeng. 2001;91(4):390-5.
14. Freitas AR, Tedim AP, Francia MV, Jensen LB, Novais C, Peixe L, et al. Multilevel population genetic analysis of vanA and vanB Enterococcus faecium causing nosocomial outbreaks in 27 countries (1986-2012). J Antimicrob Chemother. 2016;71(12):3351-66.
15. Cintas LM, Casaus P, Havarstein LS, Hernandez PE, Nes IF. Biochemical and genetic characterization of enterocin P, a novel sec-dependent bacteriocin from Enterococcus faecium P13 with a broad antimicrobial spectrum. Appl Environ Microbiol. 1997;63(11):4321-30.
16. Cintas LM, Casaus P, Herranz C, Havarstein LS, Holo H, Hernandez PE, et al. Biochemical and genetic evidence that Enterococcus faecium L50 produces enterocins L50A and L50B, the sec-dependent enterocin P, and a novel bacteriocin secreted without an N-terminal extension termed enterocin Q. J Bacteriol. 2000;182(23):6806-14.
17. Herranz C, Mukhopadhyay S, Casaus P, Martinez JM, Rodriguez JM, Nes IF, et al. Biochemical and genetic evidence of enterocin P production by two Enterococcus faecium-like strains isolated from fermented sausages. Curr Microbiol. 1999;39(5):282-90.
18. Miceli de Farias F, Silva Francisco M, Nascimento de Sousa Santos I, Salustiano Marques-Bastos SL, Lemos Miguel MA, Mattos Albano R, et al. Draft genome sequence of Enterococcus faecium E86, a strain producing broad-spectrum antimicrobial peptides: Description of a novel bacteriocin immunity protein and a novel sequence type. J Glob Antimicrob Resist. 2019;17:195-7.
19. Todokoro D, Tomita H, Inoue T, Ike Y. Genetic analysis of bacteriocin 43 of vancomycin-resistant Enterococcus faecium. Appl Environ Microbiol. 2006;72(11):6955-64.
20. del Campo R, Tenorio C, Jimenez-Diaz R, Rubio C, Gomez-Lus R, Baquero F, et al. Bacteriocin production in vancomycin-resistant and vancomycin-susceptible Enterococcus isolates of different origins. Antimicrob Agents Chemother. 2001;45(3):905-12.
21. Tomita H, Fujimoto S, Tanimoto K, Ike Y. Cloning and genetic organization of the bacteriocin 31 determinant encoded on the Enterococcus faecalis pheromone-responsive conjugative plasmid pYI17. J Bacteriol. 1996;178(12):3585-93.
22. Kang JH, Lee MS. Characterization of a bacteriocin produced by Enterococcus faecium GM-1 isolated from an infant. J Appl Microbiol. 2005;98(5):1169-76.
23. Birri DJ, Brede DA, Forberg T, Holo H, Nes IF. Molecular and genetic characterization of a novel bacteriocin locus in Enterococcus avium isolates from infants. Appl Environ Microbiol. 2010;76(2):483-92.
24. De Kwaadsteniet M, Fraser T, Van Reenen CA, Dicks LM. Bacteriocin T8, a novel class IIa sec-dependent bacteriocin produced by Enterococcus faecium T8, isolated from vaginal secretions of children infected with human immunodeficiency virus. Appl Environ Microbiol. 2006;72(7):4761-6.
25. Fabricius L, Dahlen G, Holm SE, Moller AJ. Influence of combinations of oral bacteria on periapical tissues of monkeys. Scand J Dent Res. 1982;90(3):200-6.
26. Flannagan SE, Clewell DB, Sedgley CM. A "retrocidal" plasmid in Enterococcus faecalis: passage and protection. Plasmid. 2008;59(3):217-30.

27. Sedgley CM, Clewell DB, Flannagan SE. Plasmid pAMS1-encoded, bacteriocin-related "Siblicide" in Enterococcus faecalis. J Bacteriol. 2009;191(9):3183-8.
28. Sanchez J, Basanta A, Gomez-Sala B, Herranz C, Cintas LM, Hernandez PE. Antimicrobial and safety aspects, and biotechnological potential of bacteriocinogenic enterococci isolated from mallard ducks (Anas platyrhynchos). Int J Food Microbiol. 2007;117(3):295-305.
29. Sanchez J, Diep DB, Herranz C, Nes IF, Cintas LM, Hernandez PE. Amino acid and nucleotide sequence, adjacent genes, and heterologous expression of hiracin JM79, a sec-dependent bacteriocin produced by Enterococcus hirae DCH5, isolated from Mallard ducks (Anas platyrhynchos). FEMS Microbiol Lett. 2007;270(2):227-36.
30. Belguesmia Y, Choiset Y, Prevost H, Dalgalarrondo M, Chobert JM, Drider D. Partial purification and characterization of the mode of action of enterocin S37: a bacteriocin produced by Enterococcus faecalis S37 isolated from poultry feces. J Environ Public Health. 2010;2010:986460.
31. Svetoch EA, Eruslanov BV, Perelygin VV, Mitsevich EV, Mitsevich IP, Borzenkov VN, et al. Diverse antimicrobial killing by Enterococcus faecium E 50-52 bacteriocin. J Agric Food Chem. 2008;56(6):1942-8.
32. Saavedra L, Minahk C, de Ruiz Holgado AP, Sesma F. Enhancement of the enterocin CRL35 activity by a synthetic peptide derived from the NH2-terminal sequence. Antimicrob Agents Chemother. 2004;48(7):2778-81.
33. Farias ME, AAP DERH, Sesma F. Bacteriocin Production by Lactic Acid Bacteria Isolated from Regional Cheeses: Inhibition of Foodborne Pathogens. J Food Prot. 1994;57(11):1013-5.
34. Zendo T, Eungruttanagorn N, Fujioka S, Tashiro Y, Nomura K, Sera Y, et al. Identification and production of a bacteriocin from Enterococcus mundtii QU 2 isolated from soybean. J Appl Microbiol. 2005;99(5):1181-90.
35. Bennik MH, Vanloo B, Brasseur R, Gorris LG, Smid EJ. A novel bacteriocin with a YGNGV motif from vegetable-associated Enterococcus mundtii: full characterization and interaction with target organisms. Biochim Biophys Acta. 1998;1373(1):47-58.
36. Du L, Somkuti GA, Renye Jr JA, Huo G. Properties of Durancin Gl, a New Antilisterial Bacteriocin Produced by Enterococcus Durans 41d. Journal of Food Safety. 2012;32(1):74-83.
37. Renye JA, Jr., Somkuti GA, Paul M, Van Hekken DL. Characterization of antilisterial bacteriocins produced by Enterococcus faecium and Enterococcus durans isolates from Hispanic-style cheeses. J Ind Microbiol Biotechnol. 2009;36(2):261-8.
38. Kawamoto S, Shima J, Sato R, Eguchi T, Ohmomo S, Shibato J, et al. Biochemical and genetic characterization of mundticin KS, an antilisterial peptide produced by Enterococcus mundtii NFRI 7393. Appl Environ Microbiol. 2002;68(8):3830-40.
39. Eguchi T, Kaminaka K, Shima J, Kawamoto S, Mori K, Choi SH, et al. Isolation and characterization of enterocin SE-K4 produced by thermophilic enterococci, Enterococcus faecalis K-4. Biosci Biotechnol Biochem. 2001;65(2):247-53.
40. Marekova M, Laukova A, Skaugen M, Nes I. Isolation and characterization of a new bacteriocin, termed enterocin M, produced by environmental isolate Enterococcus faecium AL41. J Ind Microbiol Biotechnol. 2007;34(8):533-7.
41. Jimenez E, Delgado S, Fernandez L, Garcia N, Albujar M, Gomez A, et al. Assessment of the bacterial diversity of human colostrum and screening of staphylococcal and enterococcal populations for potential virulence factors. Res Microbiol. 2008;159(9-10):595-601.
42. Maldonado-Barragan A, Caballero-Guerrero B, Jimenez E, Jimenez-Diaz R, Ruiz-Barba JL, Rodriguez JM. Enterocin C, a class IIb bacteriocin produced by E. faecalis C901, a strain isolated from human colostrum. Int J Food Microbiol. 2009;133(1-2):105-12.

43. Balla E, Dicks LM, Du Toit M, Van Der Merwe MJ, Holzapfel WH. Characterization and cloning of the genes encoding enterocin 1071A and enterocin 1071B, two antimicrobial peptides produced by Enterococcus faecalis BFE 1071. Appl Environ Microbiol. 2000;66(4):1298-304.
44. Franz CM, Grube A, Herrmann A, Abriouel H, Starke J, Lombardi A, et al. Biochemical and genetic characterization of the two-peptide bacteriocin enterocin 1071 produced by Enterococcus faecalis FAIR-E 309. Appl Environ Microbiol. 2002;68(5):2550-4.
45. Hu CB, Malaphan W, Zendo T, Nakayama J, Sonomoto K. Enterocin X, a novel two-peptide bacteriocin from Enterococcus faecium KU-B5, has an antibacterial spectrum entirely different from those of its component peptides. Appl Environ Microbiol. 2010;76(13):4542-5.
46. Gonzalez C, Langdon GM, Bruix M, Galvez A, Valdivia E, Maqueda M, et al. Bacteriocin AS-48, a microbial cyclic polypeptide structurally and functionally related to mammalian NK-lysin. Proc Natl Acad Sci U S A. 2000;97(21):11221-6.
47. Martinez-Bueno M, Maqueda M, Galvez A, Samyn B, Van Beeumen J, Coyette J, et al. Determination of the gene sequence and the molecular structure of the enterococcal peptide antibiotic AS-48. J Bacteriol. 1994;176(20):6334-9.
48. Galvez A, Maqueda M, Valdivia E, Quesada A, Montoya E. Characterization and partial purification of a broad spectrum antibiotic AS-48 produced by Streptococcus faecalis. Can J Microbiol. 1986;32(10):765-71.
49. Tomita H, Fujimoto S, Tanimoto K, Ike Y. Cloning and genetic and sequence analyses of the bacteriocin 21 determinant encoded on the Enterococcus faecalis pheromone-responsive conjugative plasmid pPD1. J Bacteriol. 1997;179(24):7843-55.
50. Joosten HM, Nunez M, Devreese B, Van Beeumen J, Marugg JD. Purification and characterization of enterocin 4, a bacteriocin produced by Enterococcus faecalis INIA 4. Appl Environ Microbiol. 1996;62(11):4220-3.
51. Dezwaan DC, Mequio MJ, Littell JS, Allen JP, Rossbach S, Pybus V. Purification and characterization of enterocin 62-6, a two-peptide bacteriocin produced by a vaginal strain of Enterococcus faecium: Potential significance in bacterial vaginosis. Microb Ecol Health Dis. 2007;19(4):241-50.
52. Kelly MC, Mequio MJ, Pybus V. Inhibition of vaginal lactobacilli by a bacteriocin-like inhibitor produced by Enterococcus faecium 62-6: potential significance for bacterial vaginosis. Infect Dis Obstet Gynecol. 2003;11(3):147-56.
53. Al Atya AK, Drider-Hadiouche K, Ravallec R, Silvain A, Vachee A, Drider D. Probiotic potential of Enterococcus faecalis strains isolated from meconium. Front Microbiol. 2015;6(227):227.
54. Gupta H, Malik RK, Bhardwaj A, Kaur G, De S, Kaushik JK. Purification and characterization of enterocin FH 99 produced by a faecal isolate Enterococcus faecium FH 99. Indian J Microbiol. 2010;50(2):145-55.
55. Cintas LM, Casaus P, Holo H, Hernandez PE, Nes IF, Havarstein LS. Enterocins L50A and L50B, two novel bacteriocins from Enterococcus faecium L50, are related to staphylococcal hemolysins. J Bacteriol. 1998;180(8):1988-94.
56. Izquierdo E, Bednarczyk A, Schaeffer C, Cai Y, Marchioni E, Van Dorsselaer A, et al. Production of enterocins L50A, L50B, and IT, a new enterocin, by Enterococcus faecium IT62, a strain isolated from Italian ryegrass in Japan. Antimicrob Agents Chemother. 2008;52(6):1917-23.
57. Floriano B, Ruiz-Barba JL, Jimenez-Diaz R. Purification and genetic characterization of enterocin I from Enterococcus faecium 6T1a, a novel antilisterial plasmid-encoded bacteriocin which does not belong to the pediocin family of bacteriocins. Appl Environ Microbiol. 1998;64(12):4883-90.
58. Achemchem F, Martinez-Bueno M, Abrini J, Valdivia E, Maqueda M. Enterococcus faecium F58, a bacteriocinogenic strain naturally occurring in Jben, a soft, farmhouse goat's cheese made in Morocco. J Appl Microbiol. 2005;99(1):141-50.

59. Moreno MR, Leisner JJ, Tee LK, Ley C, Radu S, Rusul G, et al. Microbial analysis of Malaysian tempeh, and characterization of two bacteriocins produced by isolates of Enterococcus faecium. J Appl Microbiol. 2002;92(1):147-57.
60. Yamamoto Y, Togawa Y, Shimosaka M, Okazaki M. Purification and characterization of a novel bacteriocin produced by Enterococcus faecalis strain RJ-11. Appl Environ Microbiol. 2003;69(10):5746-53.
61. Liu X, Vederas JC, Whittal RM, Zheng J, Stiles ME, Carlson D, et al. Identification of an N-terminal formylated, two-peptide bacteriocin from Enterococcus faecalis 710C. J Agric Food Chem. 2011;59(10):5602-8.
62. Martin-Platero AM, Valdivia E, Ruiz-Rodriguez M, Soler JJ, Martin-Vivaldi M, Maqueda M, et al. Characterization of antimicrobial substances produced by Enterococcus faecalis MRR 10-3, isolated from the uropygial gland of the hoopoe (Upupa epops). Appl Environ Microbiol. 2006;72(6):4245-9.
63. Galvez A, Valdivia E, Abriouel H, Camafeita E, Mendez E, Martinez-Bueno M, et al. Isolation and characterization of enterocin EJ97, a bacteriocin produced by Enterococcus faecalis EJ97. Arch Microbiol. 1998;171(1):59-65.
64. Sanchez-Hidalgo M, Maqueda M, Galvez A, Abriouel H, Valdivia E, Martinez-Bueno M. The genes coding for enterocin EJ97 production by Enterococcus faecalis EJ97 are located on a conjugative plasmid. Appl Environ Microbiol. 2003;69(3):1633-41.
65. Reinseth I, Tonnesen HH, Carlsen H, Diep DB. Exploring the Therapeutic Potenital of the Leaderless Enterocins K1 and EJ97 in the Treatment of Vancomycin-Resistant Enterococcal Infection. Front Microbiol. 2021;12:649339.
66. Inoue T, Tomita H, Ike Y. Bac 32, a novel bacteriocin widely disseminated among clinical isolates of Enterococcus faecium. Antimicrob Agents Chemother. 2006;50(4):1202-12.
67. Yamashita H, Tomita H, Inoue T, Ike Y. Genetic organization and mode of action of a novel bacteriocin, bacteriocin 51: determinant of VanA-type vancomycin-resistant Enterococcus faecium. Antimicrob Agents Chemother. 2011;55(9):4352-60.
68. Phumisantiphong U, Siripanichgon K, Reamtong O, Diraphat P. A novel bacteriocin from Enterococcus faecalis 478 exhibits a potent activity against vancomycin-resistant enterococci. PLoS One. 2017;12(10):e0186415.
69. Izquierdo E, Wagner C, Marchioni E, Aoude-Werner D, Ennahar S. Enterocin 96, a novel class II bacteriocin produced by Enterococcus faecalis WHE 96, isolated from Munster cheese. Appl Environ Microbiol. 2009;75(13):4273-6.
70. Maky MA, Ishibashi N, Zendo T, Perez RH, Doud JR, Karmi M, et al. Enterocin F4-9, a Novel O-Linked Glycosylated Bacteriocin. Appl Environ Microbiol. 2015;81(14):4819-26.
71. Hanchi H, Hammami R, Gingras H, Kourda R, Bergeron MG, Ben Hamida J, et al. Inhibition of MRSA and of Clostridium difficile by durancin 61A: synergy with bacteriocins and antibiotics. Future Microbiol. 2017;12:205-12.
72. Izquierdo E, Cai Y, Marchioni E, Ennahar S. Genetic identification of the bacteriocins produced by Enterococcus faecium IT62 and evidence that bacteriocin 32 is identical to enterocin IT. Antimicrob Agents Chemother. 2009;53(5):1907-11.
73. Kang BS, Seo JG, Lee GS, Kim JH, Kim SY, Han YW, et al. Antimicrobial activity of enterocins from Enterococcus faecalis SL-5 against Propionibacterium acnes, the causative agent in acne vulgaris, and its therapeutic effect. J Microbiol. 2009;47(1):101-9.
74. Nilsen T, Nes IF, Holo H. Enterolysin A, a cell wall-degrading bacteriocin from Enterococcus faecalis LMG 2333. Appl Environ Microbiol. 2003;69(5):2975-84.
75. Hickey RM, Twomey DP, Ross RP, Hill C. Production of enterolysin A by a raw milk enterococcal isolate exhibiting multiple virulence factors. Microbiology (Reading). 2003;149(Pt 3):655-64.

76. Tomita H, Kamei E, Ike Y. Cloning and genetic analyses of the bacteriocin 41 determinant encoded on the Enterococcus faecalis pheromone-responsive conjugative plasmid pYI14: a novel bacteriocin complemented by two extracellular components (lysin and activator). J Bacteriol. 2008;190(6):2075-85.
77. Laukova A, Czikkova S. The use of enterocin CCM 4231 in soy milk to control the growth of Listeria monocytogenes and Staphylococcus aureus. J Appl Microbiol. 1999;87(1):182-6.
78. Lauková A, Mareková M, Javorský P. Detection and antimicrobial spectrum of a bacteriocin-like substance produced by Enterococcus faecium CCM4231. Letters in Applied Microbiology. 1993;16(5):257-60.
79. Sabia C, de Niederhausern S, Messi P, Manicardi G, Bondi M. Bacteriocin-producing Enterococcus casseliflavus IM 416K1, a natural antagonist for control of Listeria monocytogenes in Italian sausages ("cacciatore"). Int J Food Microbiol. 2003;87(1-2):173-9.
80. Huang E, Zhang L, Chung YK, Zheng Z, Yousef AE. Characterization and application of enterocin RM6, a bacteriocin from Enterococcus faecalis. Biomed Res Int. 2013;2013:206917.
